# Autologous Peripheral Vγ9Vδ2 T Cell Synergizes with αβ T Cell Through Antigen Presentation and BTN3A1 Blockade in Immunotherapy of Cervical Cancer

**DOI:** 10.1002/advs.202401230

**Published:** 2025-03-17

**Authors:** Min Wu, Jian Liu, Liting Liu, Yifan Yang, Hong Liu, Long Yu, Haihong Zeng, Shuo Yuan, Ruiyi Xu, Hangyu Liu, Han Jiang, Shen Qu, Liming Wang, Ying Chen, Jingyu Wang, Yuwei Zhang, Shan He, Ling Feng, Junyan Han, Wanjiang Zeng, Hui Wang, Yafei Huang

**Affiliations:** ^1^ Department of Obstetrics and Gynecology, Tongji Hospital and School of Basic Medicine, Tongji Medical College Huazhong University of Science and Technology Wuhan 430030 China; ^2^ Department of Gynecologic Oncology, Women's Hospital Zhejiang University School of Medicine Hangzhou Zhejiang 310006 China; ^3^ Beckman Coulter Commercial Enterprise (China) Co., Ltd Shanghai 200122 China; ^4^ Department of Pathogen Biology, School of Basic Medicine, Tongji Medical College Huazhong University of Science and Technology Wuhan 430030 China; ^5^ Department of Immunology, School of Basic Medicine, Tongji Medical College Huazhong University of Science and Technology Wuhan 430030 China; ^6^ Department of Obstetrics and Gynecology, Tongji Hospital Tongji Medical College Huazhong University of Science and Technology Wuhan 430030 China; ^7^ Department of Obstetrics and Gynecology Tongji Hospital, Tongji Medical College, Huazhong University of Science and Technology Cancer Biology Research Center (Key Laboratory of the Ministry of Education), Tongji Hospital, Tongji Medical College Huazhong University of Science and Technology Wuhan China; ^8^ Department of Gynecologic Oncology, Women's Hospital Zhejiang University School of Medicine Zhejiang Provincial Key Laboratory of Precision Diagnosis and Therapy for Major Gynecological Diseases, Women's Hospital Zhejiang University School of Medicine Hangzhou Zhejiang China; ^9^ State Key Laboratory for Diagnosis and Treatment of Severe Zoonotic Infectious Diseases Huazhong University of Science and Technology Wuhan 430030 China

**Keywords:** antigen presentation, BTN3A1, cervical cancer, immunotherapy, Vγ9Vδ2 T cell, α, αβ T cell

## Abstract

New treatment strategies are urgently needed for patients with advanced cervical cancer (CC). Here, a synergistic anti‐CC effect of a novel combinatorial immunotherapy with adoptively transferred autologous Vγ9Vδ2 T cells and αβ T cells is shown. The pivotal role of both circulating and tumor‐infiltrating Vγ9Vδ2 T cells in anti‐CC immunity is uncovered. Importantly, autologous Vγ9Vδ2 T cells show a synergistic anti‐CC effect with αβ T cells not only through killing tumor directly, but also by promoting the activation and tumoricidal activity of syngeneic αβ T cells through antigen presentation, which can be further boosted by conventional chemotherapy. Moreover, Vγ9Vδ2 T cells can restore the tumoricidal function of αβ T cell through competitively binding to BTN3A1, a TCR‐Vγ9Vδ2 ligand on CC cells upregulated by IFN‐γ derived from activated αβ T cell. These findings uncover a critical synergistic effect of autologous Vγ9Vδ2 T cells and αβ T cells in immunotherapy of CC and reveal the underlying mechanisms.

## Graphical abstract

This study provides proof‐of‐concept evidence showing that through direct killing, antigen presentation, and competitively binding to BTN3A1, Vγ9Vδ2 T cells amplified from cervical cancer (CC) patients can synergize with αβ T cells to exert anti‐CC function both in vitro and in vivo. Furthermore, this novel combinatorial therapy can be combined with common chemotherapy.

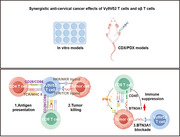

## 1 Introduction

Cervical cancer (CC) is the fourth most common cancer in women, with 604127 new cases and 341831 deaths registered worldwide in 2020.^[^ ^1^ ^]^ Although early‐stage CC is mostly curable, advanced CC has a poor prognosis due to the lack of effective treatments, with the overall survival (OS) and 5‐year survival rates of only 7 months and 17%, respectively.^[^ ^2^ ^]^ Therefore, development of novel effective therapies is of utmost importance for treating this malignancy. Recently, immunotherapy has been increasingly recognized as an effective treatment modality in an array of cancers,^[^ ^3^, ^4^, ^5^ ^]^ and encouragingly, several immunotherapy strategies including immune checkpoint inhibitor (ICI),^[^ ^6^, ^7^ ^]^ therapeutic vaccines,^[^ ^8^, ^9^, ^10^ ^]^ and adoptive T cell therapy (ACT) with T cell receptor gene engineered T cells (TCR‐T)^[^ ^11^, ^12^ ^]^ and tumor infiltrating lymphocytes (TILs)^[^ ^13^, ^14^, ^15^ ^]^ have been applied and even approved in treating patients suffered from advanced CC. Despite these progresses, limited clinical success has been made as exemplified by the approved ICI therapy, the objective response rate of which as monotherapy in CC was 14.6%–27.8%, and was only improved to 22%–46% when the PD‐1 and CTLA‐4 bispecific antibody drugs or the combination of PD‐1 and CTLA‐4 monoclonal antibodies was used.^[^ ^5^, ^10^ ^]^

Most of the current immunotherapy strategies are aiming to improve the quality and quantity of αβ T cells,^[^ ^16^, ^17^ ^]^ which could specifically recognize and destroy tumors bearing target antigen. On the other hand, the optimal priming, maintenance and tumoricidal effects of antitumor αβ T cells are critically dependent on the functionality of antigen‐presenting cells (APCs) which present tumor‐derived peptide antigen in the context of major histocompatibility complex (MHC) molecule to tumor‐specific αβ T cells.^[^ ^18^, ^19^, ^20^ ^]^ Unfortunately, immune escape mechanisms such as decreased expression or heterozygosity loss of human leukocyte antigen (HLA) in tumor cell, and functional impairment in APC, are often developed in advanced CC patients,^[^ ^21^ ^]^ thereby leading to the defect in activating and maintaining the antitumor function of αβ T cell after immunotherapy.^[^ ^10^ ^]^ In contrast to αβ T cell, γδ T cell, another T lymphocyte subset that utilizes γ and δ chains to assemble its TCR heterodimer, could recognize stress/infection/cancer‐induced molecules such as MICA/B and phosphoantigens (PAg) independent of MHC molecules.^[^ ^22^, ^23^, ^24^ ^]^ This characteristic makes γδ T cell a potent anti‐tumor effector cell. This notion is supported by the finding that intratumoral γδ T cell is the most predictive immune signature of improved outcomes across 25 cancers.^[^ ^25^ ^]^ Furthermore, Vγ9Vδ2 subset, the predominant human circulating γδ T cell subpopulation that can be activated by PAg overproduced by tumor cells in a butyrophilin 3A1 (BTN3A1) dependent manner, has been frequently selected for ACT in a variety of cancers.^[^ ^22^, ^23^, ^24^, ^26^ ^]^ Additionally, Vγ9Vδ2 T cells can interact with αβ T cells, and thereby modulate immune responses through multiple mechanisms. Vγ9Vδ2 T cells have been reported to secrete cytokines such as IFN‐γ and IL‐17, which can activate or modulate αβ T cell functions. Furthermore, in the tumor microenvironment, Vγ9Vδ2 T cells are capable of synergizing with αβ T cells to enhance anti‐tumor immunity through direct cytotoxicity and immunomodulation.^[^ ^23^, ^24^ ^]^ Another noteworthy interaction is that Vγ9Vδ2 T cell can promote the activation and anti‐tumor function of αβ T cell through antigen presentation and cross‐presentation.^[^ ^24^, ^27^, ^28^, ^29^ ^]^ Therefore, understanding the interaction between Vγ9Vδ2 T cells and αβ T cells is critical for developing novel immunotherapies targeting both innate and adaptive immune pathways. However, although proposed in as early as 2014,^[^ ^30^ ^]^ to date, clinical trials aiming to use the antigen‐presenting capacity of Vγ9Vδ2 cells have not been explored. In addition, the role of these cells in anti‐CC immunity and the underlying mechanism are still not clear.

In the present study, we investigated the potential role of Vγ9Vδ2 T cells in anti‐CC immunity using clinical data, examined the synergistic anti‐CC effect of expanded autologous Vγ9Vδ2 T cells with cognate αβ T cells and chemotherapy by in vitro and in vivo experiments, and explored the underlying mechanisms. Our data establish Vγ9Vδ2 T cell as a potent anti‐CC immune cell that can synergize with αβ T cell through direct tumor‐killing, antigen presentation and relieving BTN3A1‐mediated αβ T cell inhibition. These findings shed new light on the combinatorial immunotherapy of CC with two distinct T lymphocytes.

## 2 Results

### 2.1 The Abundance of γδ TILs is Associated with Better Prognosis and Antitumor Immunity in CC

**Figure 1:**
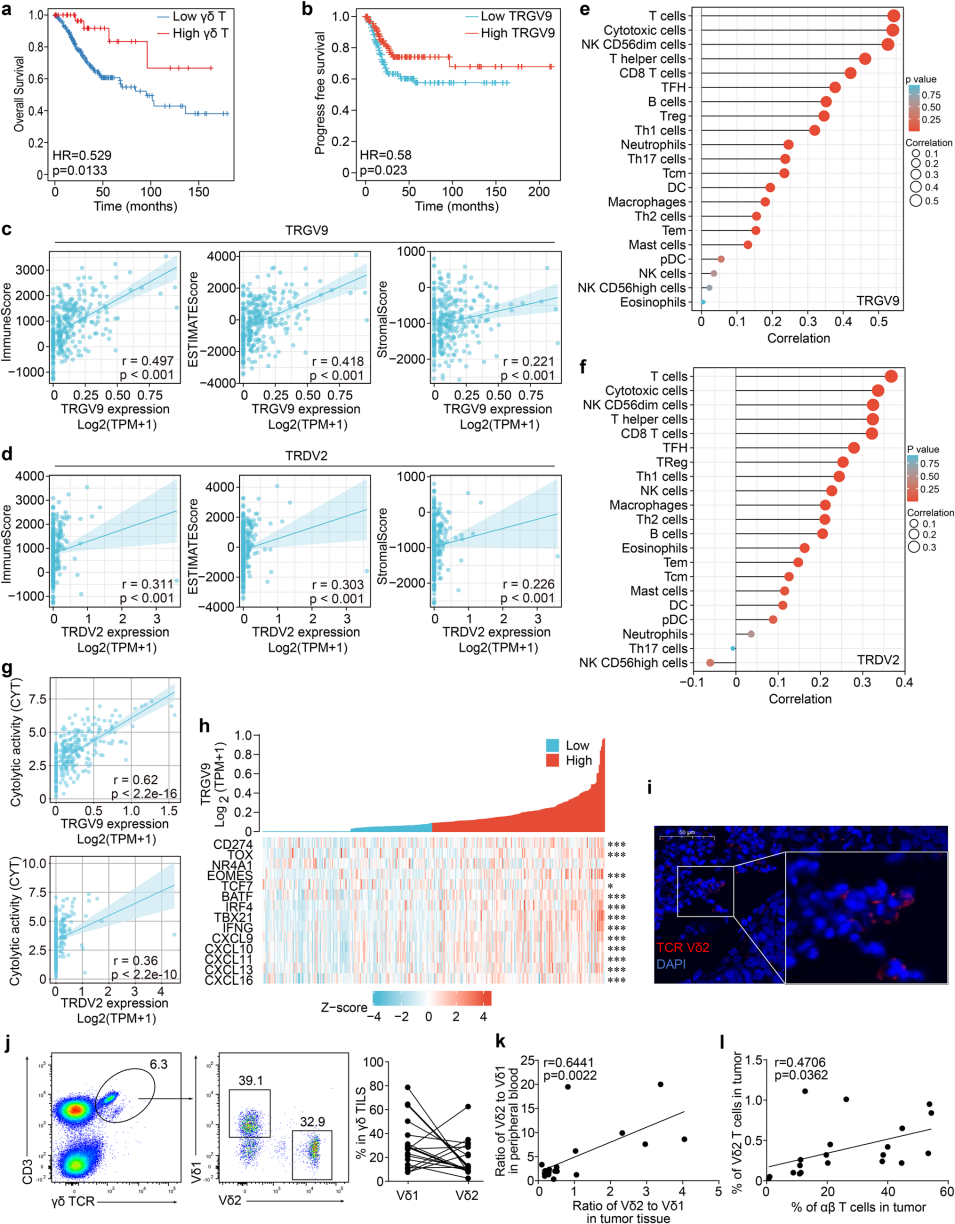
Intratumoral Vγ9Vδ2 T cell correlates with better prognosis and antitumor immunity in CC. a) Relationship of γδ T cell abundance (determined by CIBERSORT) in tumor with CC patient survival (Kaplan‐Meier survival) in TCGA dataset (n = 306, analyzed in TIMER 2.0 database; Results are shown as individual cancer survival curves with top 50% high and low γδ T cell abundance). b) Relationship of *TRGV9* expression level in tumor with CC patient survival in TCGA dataset (n = 306, Cox regression was used). c, d) The correlations of *TRGV9* (c) and *TRDV2* (d) expression levels with the indexes of ImmunoScore, ESTIMATEScore and StromalScore in CC from TCGA dataset (n = 306). Estimate algorithm [1.0.13] and spearman statistics were used. e, f) The correlations of *TRGV9* (e) and *TRDV2* (f) expression levels with the infiltration of various immune cell subsets in CC from TCGA dataset (n = 306). ssGSEA algorithm and spearman statistics were used. g) The correlations of *TRGV9* and *TRDV2* expression levels with the index of Cytolytic activity (CYT) score in CC from TCGA dataset (n = 306). Spearman statistics were used. h) The correlation of *TRGV9* expression level with the expression of molecules related to T cell activation, differentiation, chemotaxis and exhaustion in CC from TCGA dataset (n = 306). Spearman statistics were used. i) Representative immunofluorescence image showed the presence of Vδ2 γδ T subset (red fluorescence labeled cells) within CC. Scale bar: 50 µm. j) Vδ1 and Vδ2 γδ T subsets in the tumor tissues of CC patients were determined by flow cytometry. The flow cytometry plot (left) and the percentages of Vδ1 and Vδ2 γδ T subsets matched in each patient (right) were shown (n = 20). k) The correlation of frequency ratio of Vδ2 to Vδ1 subsets between tumor tissues and peripheral blood of CC patients (n = 20). Pearson correlation and two‐tailed *p* value were used. l) The correlation analysis between Vδ2 γδ T subset frequency and αβ T cell frequency in tumor tissues of CC patients (n = 20). Pearson correlation and two‐tailed *p* value were used.

Previous investigations have found that high frequency of intratumoral γδ T cells is the most significant immune signature associated with improved outcomes across 25 cancers.^[^ ^25^ ^]^ We sought to re‐examine this issue in CC in the current study. For this purpose, the abundance of intratumoral γδ T cells and the major two γδ T cell subsets, Vδ1 and Vγ9Vδ2 cells, were determined by the expression levels of their signature genes, *TRDC*, *TRDV1*, and *TRGV9*, respectively, through analyzing the RNA sequencing data of CC patients in TCGA database. Our analysis indicated that the abundance of intratumoral γδ T cells was significantly associated with increased OS of CC patients (**Figure** **1**a). Similarly, markedly better progression‐free survival (PFS) was evident in CC patients with higher infiltration of both Vδ1 cells (HR = 0.54, 95% CI 0.34–0.87, *p* = 0.011) and Vγ9Vδ2 cells (HR = 0.58, 95% CI 0.36‐0.93, *p* = 0.023) (Figure 1b; Figure S1a, Supporting Information), suggesting the antitumor effect of γδ T cells in CC. Next, we examined the relationship between tumor‐infiltrating γδ T cells (γδ TILs) and other antitumor immune cells, the latter of which was evaluated by calculating the indexes of ImmunoScore, StromalScore and ESTIMATEScore,^[^ ^31^ ^]^ as well as the frequencies of individual antitumor immune cell populations in tumor tissues.^[^ ^32^ ^]^ Our results demonstrated that the frequencies of both Vγ9Vδ2 (Figure 1c–f; Figure S1b,c, Supporting Information) and Vδ1 TILs (Figure S1d,e, Supporting Information) are highly correlated with the infiltration of several other antitumor immune cells in CC tissues. Moreover, cytolytic activity (CYT) score, an index of cancer immunity that has been used for reflecting the status of antitumor immune response and as a prognostic marker,^[^ ^33^ ^]^ was found to be positively correlated with both *TRGV9* and *TRDV2* in CC tissue (Figure 1g). Furthermore, the expression of antitumor T cell activation markers, such as *PDCD1*, *EOMES*, *BATF*, *IRF4*, *T‐bet*, *IFNG*, and chemokines including *CXCL9*, *CXCL10*, *CXCL11*, *CXCL13*, and *CXCL16*, were much higher in *TRGV9* ^high^ group than that in *TRGV9* ^low^ group (Figure 1h). These results collectively indicate that Vγ9Vδ2 T cells are positively correlated with the activation, recruitment and killing capacity of antitumor T cells. Interestingly, *TRDV1* also showed a similar correlation with the expression of T cell activation markers and chemokines (Figure S1f, Supporting Information). However, only the expression level of *TRGV9*, but not *TRDV1*, was correlated with *TCF7*, a stem‐like T cell marker in tumor tissues^[^ ^34^, ^35^ ^]^ (Figure 1h; Figure S1f, Supporting Information).

To verify these results, we examined γδ TILs in CC patients by flow cytometry. In healthy adults, Vγ9Vδ2 T cells are the most abundant γδ T cell subset in periphery, and in contrast are the minority in tissue where Vδ1 T cells predominant.^[^ ^24^, ^36^ ^]^ Interestingly, we found a high infiltration of Vγ9Vδ2 T cells in some CC tissues (Figure 1i,j). Indeed, the proportion of Vγ9Vδ2 T cells in all γδ TILs and the ratio of Vγ9Vδ2 to Vδ1 in tumor tissue can be as high as 60% and 4:1, respectively (Figure 1j,k). Importantly, when the association between the infiltration of γδ TIL subsets and αβ TILs was determined, only the frequency of Vγ9Vδ2 TILs (Figure 1l, p = 0.0362) but not Vδ1 TILs (Figure S1g, Supporting Information, *p* = 0.07) was found to be significantly associated with αβ T cell abundance.

Taken together, these results suggest that Vγ9Vδ2 TILs, and to a lesser extent, Vδ1 TILs, are associated with the better prognosis of patients as well as the infiltration and activation of antitumor αβ T cells in CC.

### 2.2 Peripheral Vγ9Vδ2 T Cells from CC Patients are Higher in Proportion and Antitumor Potential than those from Healthy Controls (HC)

**Figure 2:**
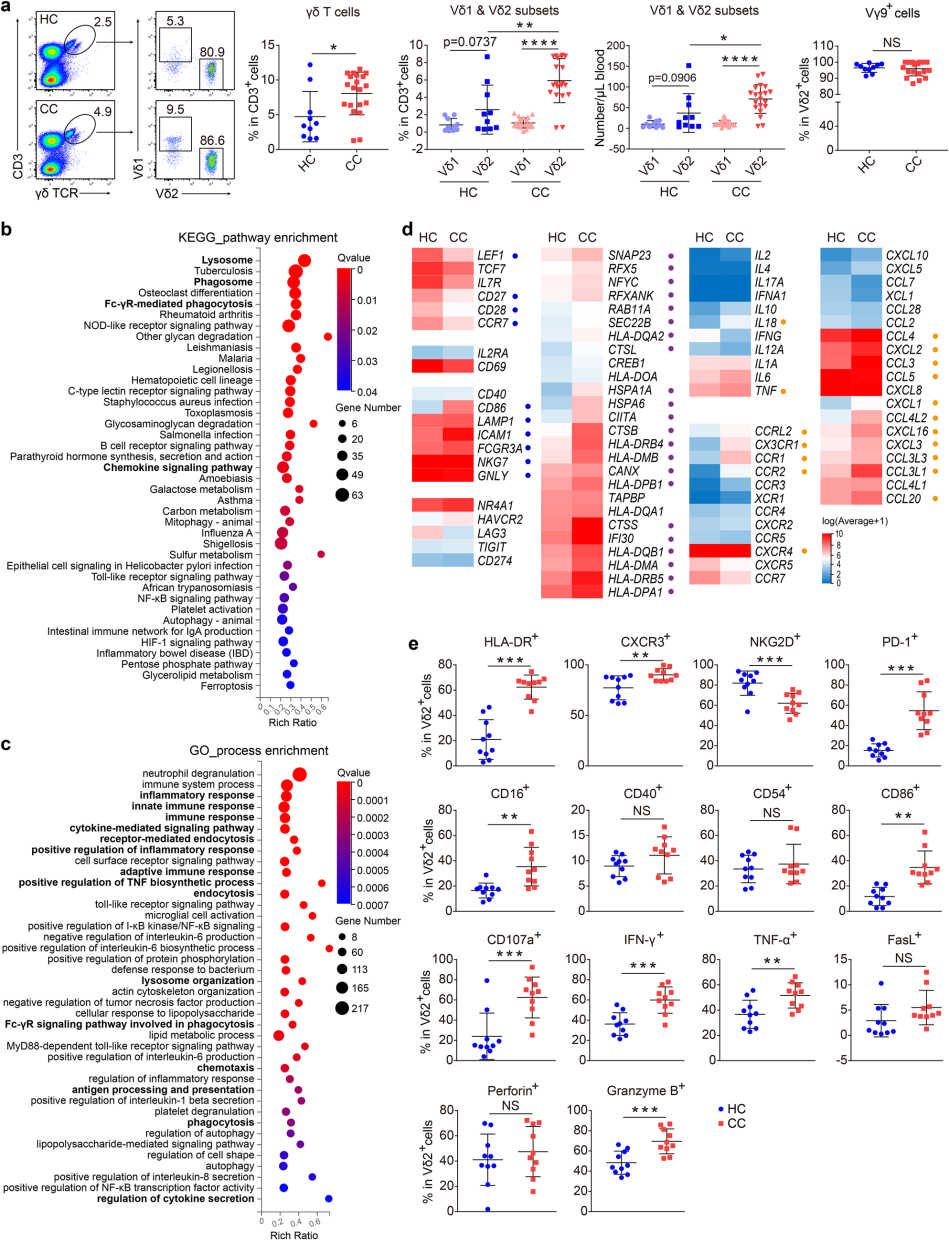
Peripheral Vγ9Vδ2 T cells from CC patients show higher proportion, activation and antigen presentation potential than those from HC. a) Peripheral γδ T cells, Vδ1, Vδ2 and Vγ9 γδ T subsets in CC patients were determined by flow cytometry. The flow cytometry plot (left) and the percentages and numbers of each subset (right) were shown. HC, n = 10. CC, n = 20. b) KEGG enrichment analysis of the up‐regulated genes in pVγ9Vδ2 T cells from CC patients compared with those from HC subjects. n = 2 per group. c) GO enrichment analysis of the up‐regulated genes in pVγ9Vδ2 T cells from CC patients compared with those from healthy people. n = 2 per group. d) The transcription levels of molecules related to T cell activation, differentiation, killing, antigen presentation, exhaustion and cytokines, chemokines and chemokine receptors in pVγ9Vδ2 T cells from CC patients and HC subjects. n = 2 per group. e) The protein expression levels (percentages) of the molecules related to T cell activation, killing, antigen presentation, exhaustion and cytokines in pVγ9Vδ2 T cells from CC and HC. n = 10 per group. Error bars represent mean with SD in (a) and (e). *p* values were calculated using two‐tailed Student's *t* test in (a) and (e). *p < 0.05; **p < 0.01; ***p < 0.001; ****p < 0.0001. NS, Not Significant.

Next, we sought to determine whether peripheral Vγ9Vδ2 (pVγ9Vδ2) T cell, which has been frequently expanded for tumor immunotherapy,^[^ ^24^ ^]^ is also equipped with antitumor potential in CC patients. Indeed, the Vδ2: Vδ1 ratio in peripheral was positively correlated with that in tumor tissue in CC patients (Figure 1k), indicating that pVγ9Vδ2 T cell is partially reflective of its tumor‐infiltrating counterpart. Furthermore, CC patients had significantly more total γδ T cells and Vγ9Vδ2 T cells, but not Vδ1 T cells in peripheral blood, than HC subjects (**Figure** **2**a). Moreover, pVγ9Vδ2 T cell from CC patients skewed more into a memory phenotype compared to that from HC subjects (Figure S2a, Supporting Information), suggesting that pVγ9Vδ2 T cell might play a role in CC.

To further explore the difference between CC‐ and HC‐derived pVγ9Vδ2 T cell, the transcriptional profiles of purified pVγ9Vδ2 T cell from these two groups were analyzed after RNA sequencing. Differentially expressed genes (DEGs) were subjected to KEGG and GO enrichment analyses, which revealed that the up‐regulated genes of pVγ9Vδ2 T cell from CC patients, when compared with those from HC, were enriched in pathways including immune response, lysosome, phagosome, phagocytosis, endocytosis, chemotaxis, cytokine secretion, and antigen processing and presentation (Figure 2b,c; Figure S2b,c, Supporting Information). This finding was further supported by direct comparison of individual DEGs between the two groups, which showed that compared to HC‐derived pVγ9Vδ2 cell, CC‐derived counterpart had increased potential of activation and differentiation levels (blue circles labeled genes), cytokine and chemokine production, chemotaxis (yellow circles labeled genes), and antigen presentation and cross‐presentation capacity (purple circles labeled genes) (Figure 2d). Vγ9Vδ2 T cells have been reported to have a high antigen‐presenting capacity comparable to dendritic cells (DC),^[^ ^27^ ^]^ the most potent professional APC. Thus, to further explore the antigen‐presentation potential of CC‐derived pVγ9Vδ2 T cell, we took DC as a reference. By using GSE database (GSE3982), DEGs enriched in DC compared to other immune cells were identified and were therefore called DC‐specific DEGs, which were used to reflect the antigen‐presenting capacity of a certain cell population, in this case, pVγ9Vδ2 T cell. This analysis revealed that these DC‐specific DEGs were mainly enriched in CC‐derived, but not HC‐derived pVγ9Vδ2 T cell (Figure S2d, Supporting Information), further elaborating the notion that CC‐derived pVγ9Vδ2 T cell has superior antigen‐presenting potential to HC‐derived counterparts.

We next verified the antitumor and antigen‐presentation potential of pVγ9Vδ2 T cell at the protein level. Again, compared with HC‐derived counterpart, CC‐derived pVγ9Vδ2 T cell expressed higher levels of HLA‐DR, CXCR3, PD‐1, CD16, CD86, CD107a, IFN‐γ, TNF‐α, and granzyme B (Figure 2e; Figure S2e, Supporting Information).

Together, these results indicate that pVγ9Vδ2 T cells of CC patients display a higher proportion, and increased potential of activation, chemotaxis, killing and antigen presentation than that of HC subjects.

### 2.3 Expanded CC Derived pVγ9Vδ2 T Cells Show a Strong Antitumor Phenotype

**Figure 3:**
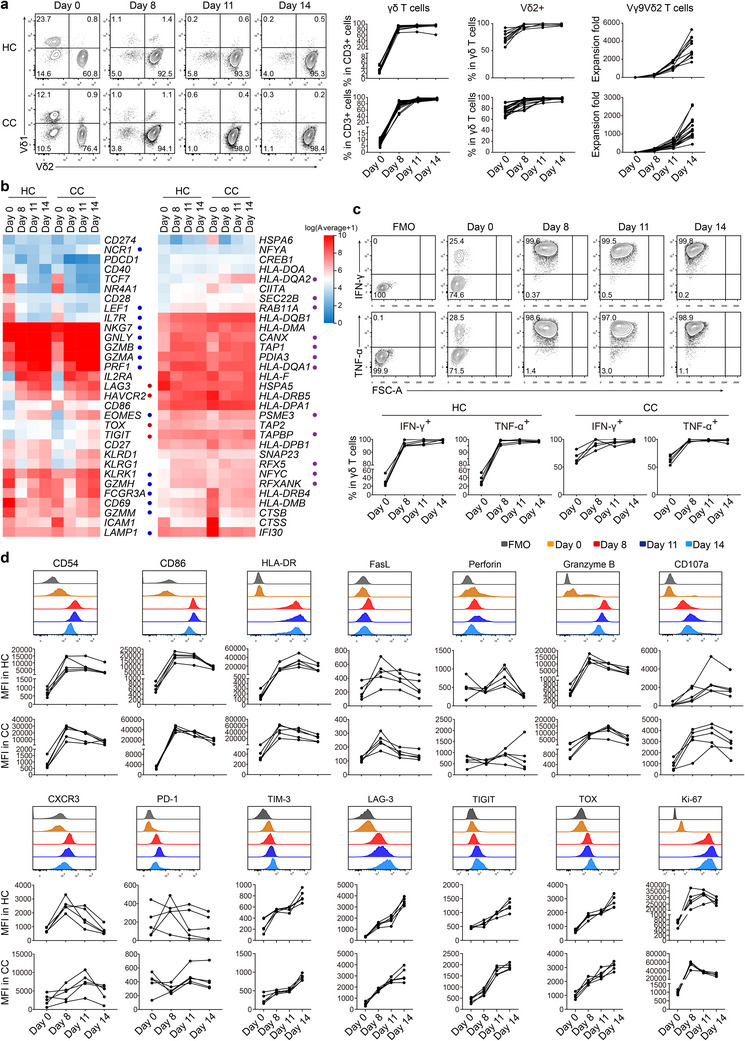
Peripheral Vγ9Vδ2 T cells of CC patients maintain antitumor phenotype after in vitro expansion. a) The percentages and expansion folds of peripheral γδ T cells and Vγ9Vδ2 subset from CC patients and HC subjects before and after in vitro expansion. HC, n = 10. CC, n = 20. b) The transcriptional levels of molecules related to T cell activation, differentiation, killing, exhaustion and antigen presentation in CC‐ and HC‐derived pVγ9Vδ2 T cells before and after expansion for indicated days. n = 2 per group. c) The protein expression levels of IFN‐γ and TNF‐α in CC‐ and HC‐derived pVγ9Vδ2 T cells before and after expansion for indicated days. n = 5 per group. d) The protein expression levels of molecules related to T cell activation, differentiation, killing, antigen presentation and exhaustion in CC and HC derived pVγ9Vδ2 T cells before and after expansion for indicated days. n = 5 per group.

The high antitumor and antigen‐presentation potential of CC‐derived pVγ9Vδ2 T cell make it an attractive candidate for CC immunotherapy. However, previous investigations have shown that patient‐derived pVγ9Vδ2 T cells are relatively hard to be expanded in some advanced cancers.^[^ ^37^ ^]^ We therefore examined this issue in CC using the classical IL‐2 and zoledronate expansion protocol.^[^ ^26^, ^38^ ^]^ In fact, all CC‐derived pVγ9Vδ2 T cells (n = 20) can be successfully expanded about several thousand folds after 14 days of expansion. Furthermore, the expansion kinetics, as assessed by counting the number of expanded cells at day 0, 8, 11, and 14 after stimulation, were comparable between CC‐ and HC‐derived pVγ9Vδ2 T cells (n = 20 and 10, respectively, **Figure** **3**a; Figure S3a, Supporting Information). Of note, after 11 and 14 days of expansion, cell products from both groups were mainly Vγ9Vδ2 T cells with over 90% purity (Figure 3a; Figure S3b, Supporting Information). The only difference we noted was that the expansion products from HC were mainly composed of central memory and effector memory T cells, while those from CC were primarily effector memory T cells (Figure S3c, Supporting Information).

Next, we sought to examine the functional potential of our expansion products through analyzing the transcriptomic profiles of Vγ9Vδ2 T cells before (day 0, D0) and after (D8, D11, and D14) expansion by RNA sequencing. Enhanced activation and killing potential (blue circles labeled genes), and elevated antigen presenting potential (purple circles labeled genes) were already observed in expansion products at D8 compared to those before expansion, and were further increased at D11 and D14 (Figure 3b). In addition, the D11 and D14 cell products expressed higher levels of *TNF*, *CCL3*, *CCL4*, *CXCR3* and other cytokines, chemokines and chemokine receptors (yellow circles labeled) than the D8 counterpart (Figure S3d, Supporting Information). However, the expression levels of exhaustion‐related molecules such as *LAG3*, *TIM3*, *TIGIT*, and *TOX* (red circles labeled) were higher in D14 than that in D8 and D11 cell products (Figure 3b), suggesting the functional impairment of the 14‐day expanded cell products. Interestingly, there was no significant difference between the HC and CC groups in aforementioned transcriptional profiles of the amplified Vγ9Vδ2 T cells examined at the same time points, indicating that the transcriptional convergence of pVγ9Vδ2 T cells from different sources after expansion.

We next verify the above transcriptional features at the protein level, and found that the amplified pVγ9Vδ2 T cells showed high expression of molecules related to activation, antigen presentation and killing, such as IFN‐γ, TNF‐α, CD54, CD86, HLA‐DR, NKG2D, CXCR3, FasL, perforin, granzyme B, and CD107a, and the expression levels of most of these molecules were higher in D8 and D11 than that in D14 cell products (Figure 3c,d; Figure S3e, Supporting Information). Of note, the protein expression levels of TIM‐3, LAG‐3, TIGIT, and TOX in Vγ9Vδ2 T cells were largely consistent with transcriptomic data: the expression levels of these inhibitory receptors gradually increased with prolonged expansion time. Again, the expression patterns of these molecules were almost the same between the HC and CC groups (Figure 3d). Interestingly, the expression levels of Ki‐67 in amplified pVγ9Vδ2 T cells exhibited dynamic changes. In pVγ9Vδ2 T cells derived from HC, Ki‐67 expression increased with the days of expansion, peaking on D11, and subsequently decreased, with lower expression on D14 compared to D11. In pVγ9Vδ2 T cells derived from CC patients, Ki‐67 expression peaked on D8 and gradually declined thereafter, with significantly lower levels on D14 compared to D8 and D11 (Figure 3d).

Taken together, these results indicate that CC‐ and HC‐derived pVγ9Vδ2 T cells can both be expanded up to several thousand folds in vitro while maintaining and even enhancing their antitumor potential after at least 11 days of amplification with IL‐2 and zoledronate stimulation. Importantly, the 11‐day‐expanded Vγ9Vδ2 T cells (D11 pVγ9Vδ2 T cells), except for their strong antitumor potential, also displayed a less exhaustion‐like phenotype, and were therefore selected for further validation.

### 2.4 D11 pVγ9Vδ2 T Cells Exhibit Strong Tumor Antigen Presenting Capacity

**Figure 4:**
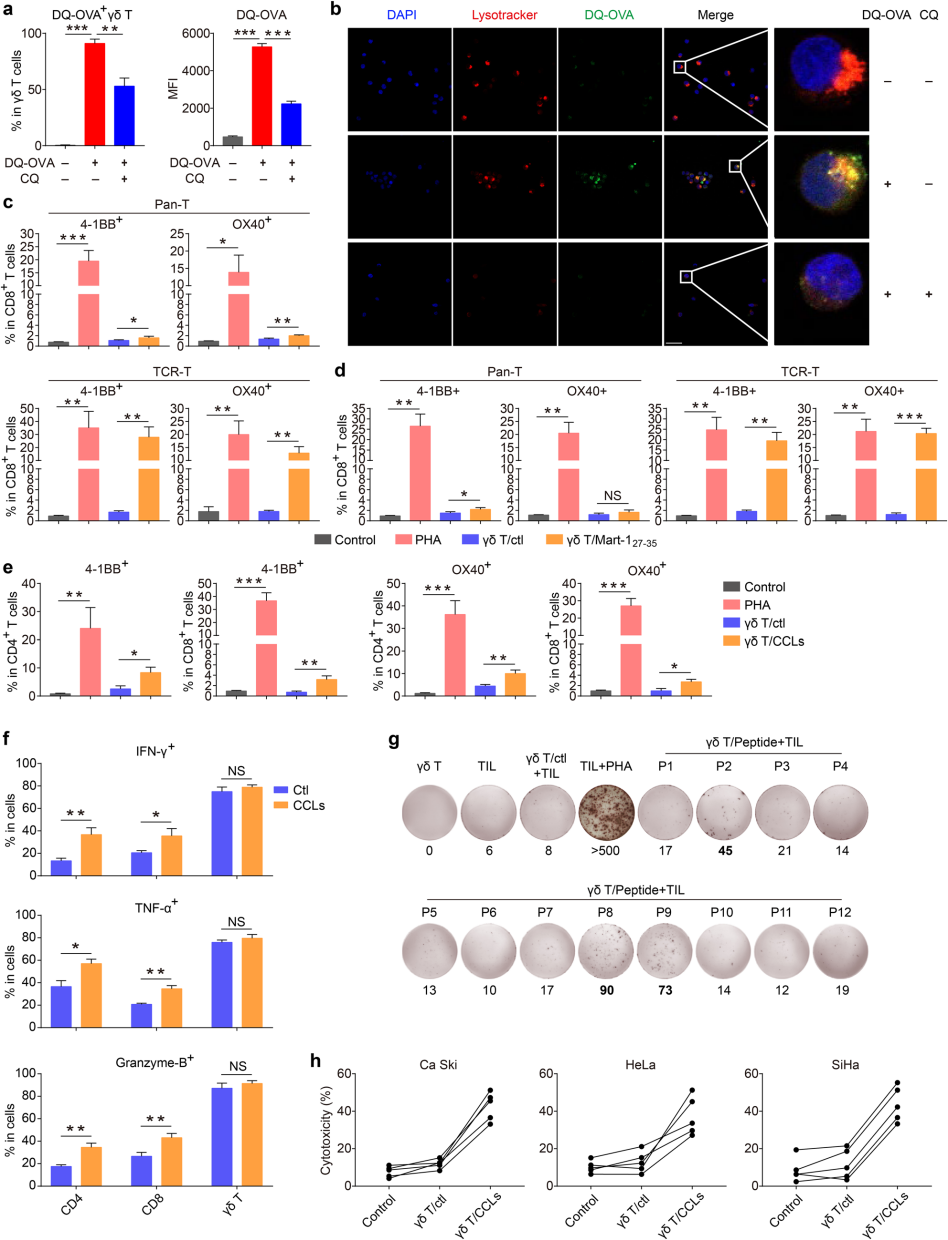
Expanded autologous pVγ9Vδ2 T cells induce the activation and tumoricidal activity of CD4^+^ and CD8^+^ αβ T cell through antigen presentation. a) Expanded pVγ9Vδ2 T cells from CC patients were cocultured with DQ‐OVA in the presence or absence of chloroquine (CQ) for 6 h. The proportion of DQ fluorescence positive cells and the intensity of DQ fluorescence were determined by flow cytometry. n = 3 independent patients. CQ, chloroquine. MFI, median fluorescence intensity. b) Expanded pVγ9Vδ2 T cells from CC patients were cocultured with DQ‐OVA in the presence or absence of CQ for 6 h. Confocal microscopy was used to observe the colocalization of intracellular DQ fluorescence and lysosomes (stained by Lysotracker). Scale bar: 20 µm. c) Mart‐1_27‐35_ specific TCR‐T cells or control T cells (Pan‐T) were cocultured with PHA, control pVγ9Vδ2 T cells, or Mart‐1_27‐35_ peptide‐loaded pVγ9Vδ2 T cells for 24 h, the proportions of 4‐1BB^+^ and OX40^+^ T cells were determined by flow cytometry. n = 3 independent patients. Pan‐T, T cells transduced with the control vector. TCR‐T, T cells transduced with Mart‐1_27‐35_ TCR vector. d) Mart‐1_27‐35_ specific TCR‐T cells or control T cells (Pan‐T) were cocultured with PHA, pVγ9Vδ2 T cells transduced with control vector or Mart‐1_27‐35_ overexpression vector for 24 h, the proportions of 4‐1BB^+^ and OX40^+^ T cells were determined by flow cytometry. n = 3 independent patients. e) Naïve αβ T cells were cocultured with control pVγ9Vδ2 T cells or CCLs‐loaded pVγ9Vδ2 T cells for 72 h, the proportions of 4‐1BB^+^ and OX40^+^ T cells were determined by flow cytometry. n = 3 independent patients. f) Naïve αβ T cells were cocultured with control pVγ9Vδ2 T cells or CCLs loaded‐pVγ9Vδ2 T cells for 120 h, the proportions of IFN‐γ^+^, TNF^+^ and Granzyme B^+^ T cells were determined by flow cytometry. n = 3 independent patients. g) αβ TILs were expanded from CC patient CAT039 and cocultured with control pVγ9Vδ2 T cells or pVγ9Vδ2 T cells loaded with predicted neoantigen single peptide (P1‐P12) for 24 h, and the abundance of released IFN‐γ was determined by ELISPOT assay. Vγ9Vδ2 T cells were irradiated (40 Gy) before coculture. P, peptide. h) Control αβ T cells or pVγ9Vδ2 T‐stimulated αβ T cells were cocultured with CaSki, HeLa or SiHa cells for 6 h, the cytotoxicity of T cells was determined by LDH assays. pVγ9Vδ2 T‐stimulated αβ T cells were prepared by coculturing naïve αβ T cells with control pVγ9Vδ2 T cells or CCLs‐loaded pVγ9Vδ2 T cells for 120 h, followed by αβ T cell purification. n = 5 independent patients. Error bars represent mean with SD. *p* values were calculated using two‐tailed Student's *t* test. *p < 0.05; **p < 0.01; ***p < 0.001. NS, Not Significant. Ctl, control. CCLs, cervical cancer cell lysates.

Next, we went on to directly examine the anti‐CC activity of D11 pVγ9Vδ2 T cells. Expanded Vγ9Vδ2 T cells have been reported to be capable of directly killing a variety of tumor cells.^[^ ^23^, ^39^ ^]^ Consistent with these findings, we also found that our D11 pVγ9Vδ2 T cells can effectively kill CC cell lines in vitro (Figure S4a, Supporting Information). Subsequently, we verified the antigen uptake and processing capacity of amplified Vγ9Vδ2 T cells toward DQ‐OVA, an antigen complex that can release fluorescence upon ingested and processed by APCs. After 6 hours of incubation, the proportion of DQ fluorescence positive cells and the intensity of DQ fluorescence were significantly increased, which can be reversed by the addition of chloroquine, a function inhibitor of lysosome (**Figure** **4**a; Figure S4b, Supporting Information). These results were supported by confocal microscopic imaging of pVγ9Vδ2 T cell, which showed that DQ fluorescence was mainly localized in the lysosomes after uptake, and chloroquine could significantly reduce the intensity of this colocalization fluorescence (Figure 4b), indicating the capacity of expanded pVγ9Vδ2 T cell to uptake and process antigen in a lysosome‐dependent way. Of note, pVγ9Vδ2 T cells still maintained the high expression levels of antigen presentation‐related molecules such as HLA‐DR, CD80, and CD86 after antigen uptake (Figure S4c, Supporting Information).

To further explore the ability of D11 pVγ9Vδ2 T cells to stimulate syngeneic αβ T cell activation through antigen presentation, pVγ9Vδ2 T cells were loaded with either Mart‐1_27‐35_ peptide or a control peptide, and then co‐cultured with Mart‐1_27‐35_‐specific CD8^+^ TCR‐T (DMF5 TCR‐T) cells. The results indicated that DMF5 TCR‐T cells can only be activated by pVγ9Vδ2 T cells loaded with Mart‐1_27‐35_ peptide, but not the control peptide, as measured by the increased expression of 4‐1BB and OX40 on the surface of TCR‐T cells (Figure 4c; Figure S4d, Supporting Information). In contrast, control T (pan‐T) cells were not activated by Vγ9Vδ2 T cells loaded with either peptide, probably due to the low frequency of Mart‐1_27‐35_‐specific T cells in periphery. These results together indicate the antigen‐presenting ability of expanded pVγ9Vδ2 T cells. Interestingly, the TCR‐T‐activating effect of pVγ9Vδ2 T cells was also evident when these cells were overexpressed with Mart‐1 (Figure 4d; Figure S4e, Supporting Information), in which peptide‐MHC complex is generated endogenously, further emphasizing the antigen‐presentation capacity of pVγ9Vδ2 T cells. In another set of experiment, pVγ9Vδ2 T cells were loaded with a more relevant tumor antigen, mixed tumor lysates prepared from a variety of CC cell lines (CCLs). The results also showed that CCLs‐loaded but not unloaded Vγ9Vδ2 T cells, can activate CD4^+^ and CD8^+^ T cells (Figure 4e; Figure S4f, Supporting Information). In addition, naïve CD4^+^ and CD8^+^ T cells were both induced to produce high levels of antitumor molecules, such as IFN‐γ, TNF‐α, and granzyme B by CCLs‐loaded pVγ9Vδ2 T cells, which themselves maintained high expression levels of these molecules after coculture (Figure 4f; Figure S5a, Supporting Information).

Tumor neoantigens that are derived from the expression products of mutated genes in tumor cells are increasingly recognized as a promising target in antitumor immunity.^[^ ^40^, ^41^ ^]^ However, the optimal priming and maintenance of neoantigen‐specific T cells rely heavily on the antigen presentation by professional APCs.^[^ ^40^, ^41^, ^42^ ^]^ Therefore, we next ask if our expanded pVγ9Vδ2 T cells can also present this type of antigen to αβ T cells. To this end, whole exon sequencing and RNA sequencing were performed on the tumor and/or peripheral blood from a patient with advanced CC,^[^ ^41^, ^43^ ^]^ based on which tumor neoantigens were predicted by TruNeo algorithm^[^ ^44^ ^]^ (Figure S6a and Table S2, Supporting Information). The predicted neoantigens were then synthesized as 12 peptides that are 29 amino acids (aa) in length with the aa residue generated by nonsynonymous mutation put in the middle, which included both MHC I and II epitopes. The immunogenicity of these candidate neoantigens was then determined by co‐incubating with either autologous EBV‐LCL cells, the most frequently used cells for validating neoantigens,^[^ ^41^ ^]^ or expanded pVγ9Vδ2 T cells, followed by inducing the activation of autologous αβ TILs. Two peptides (P2 and P8) were unanimously determined as immunogenic using either EBV‐LCL or Vγ9Vδ2 T cells as APC, whereas higher signals were observed with Vγ9Vδ2 T cells, as revealed by the number of IFN‐γ spots. Interestingly, one peptide (P9), which only generated a weak signal with EBV‐LCL, was potent in activating αβ TILs when presented by pVγ9Vδ2 T cells (Figure 4g; Figure S6b,c, Supporting Information). Thus, these results indicated that our expanded pVγ9Vδ2 T cells are potent APCs that might outcompete EBV‐LCL in both neoantigen presenting spectrum and strength.

Taken together, these results demonstrate that our 11‐day‐expanded Vγ9Vδ2 T cells are not only potent tumor killers, but also capable of processing and presenting a variety of antigens, thereby inducing the activation of antitumor αβ T cells.

### 2.5 Combinatorial Immunotherapy with pVγ9Vδ2 T Cell and αβ T Cell Effectively Inhibits CC Cell Growth In Vitro and In Vivo

To verify the capacity of our expanded pVγ9Vδ2 T cells to induce the tumor cell‐killing capacity of αβ T cells through antigen presentation, autologous naïve αβ T cells that are matched with CC cell lines (SiHa or HeLa) for at least two HLA loci were co‐cultured with control or CCLs‐loaded pVγ9Vδ2 T cells for 5 days, and were then purified (called control αβ T cell or activated αβ T cell) and tested for killing ability in vitro (Figure S5b, Supporting Information). Compared with the control, αβ T cell activated by CCLs‐loaded pVγ9Vδ2 T cells showed a significantly higher tumoricidal capacity (Figure 4h).

**Figure 5:**
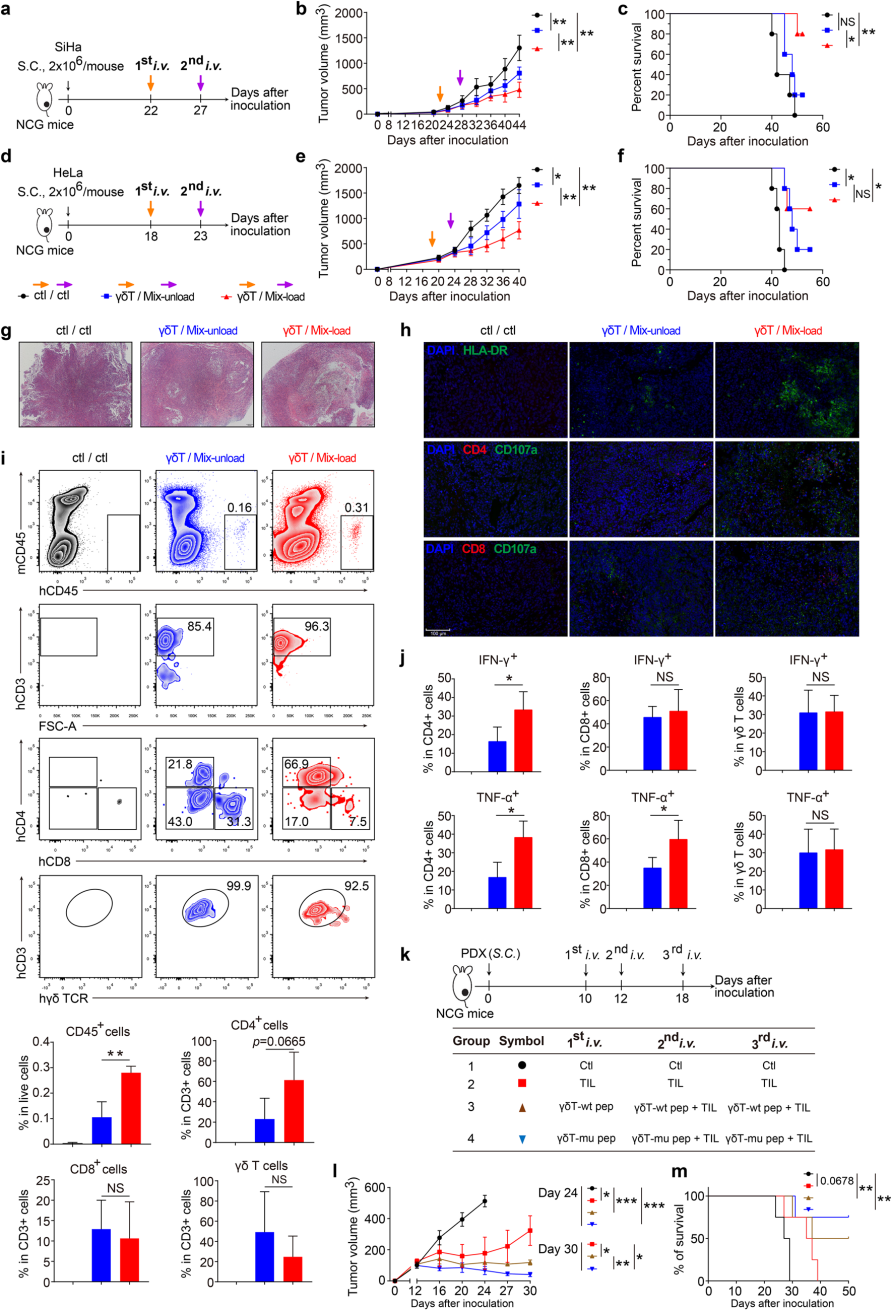
Vγ9Vδ2/αβ T cell combinatorial therapy effectively inhibits CC cell growth in vivo. a‐c) Vγ9Vδ2/αβ T cell combinatorial therapy effectively inhibits CC cell growth in a SiHa‐CDX model. SiHa cells (2 × 10^6^ cells per mouse) were inoculated subcutaneously into severely immunocompromised NCG mice. Upon tumor grew to clearly palpable (22 days after inoculation), freshly expanded pVγ9Vδ2 T cells (1 × 10^7^ cells per mouse) were injected into the tumor‐bearing mice through tail vein (1st *i.v*.). At the same time, primary αβ T cells were stimulated with equal number of autologous control or CCLs‐loaded pVγ9Vδ2 T cells to generate the control or activated αβ T cells, which were then mixed with equal number of fresh control or CCLs‐loaded pVγ9Vδ2 T cells (Mix‐unload and Mix‐load respectively), and then these cell mixtures were *i.v*. injected (27 days after inoculation) at 1 × 10^7^ cells per mouse (2nd *i.v*.). Mice that injected with medium at both injections were served as control (a). Tumor volume b) and mice survival c) were analyzed (n = 5 mice per group). The statistics in the tumor volume were calculated with the day 44 data. *S.C*., Subcutaneous. *i.v*., intravenously. d‐f) Vγ9Vδ2/αβ T cell combinatorial therapy effectively inhibits CC cell growth in a HeLa‐CDX model. Similar to that in SiHa‐CDX model (a), but 1st i.v. and 2nd i.v. were performed at day 18 and 23 after HeLa cell inoculation, respectively (d). Tumor volume (e) and mice survival f) were analyzed (n = 5 mice per group). The statistics in the tumor volume were calculated with the day 40 data. g‐j) In another set of experiments similar to a‐c, tumor‐infiltrating cells were determined at day 37 after SiHa cell inoculation. g, HE staining of tumor sections (n = 4 mice per group). h, Activated T cells (HLA‐DR^+^), CD4^+^ T cell, CD8^+^ T cell and CD107a^+^ cells in the tumor tissues were analyzed by fluorescence microscopy. Scale bar: 100 µm. i, j, Single cell suspensions of tumor tissues were prepared. Infiltration of CD45^+^ cells, CD4^+^, CD8^+^, and γδ T cells in the tumor tissues (i), and the frequencies of IFN‐γ‐ and TNF‐α‐producing CD4^+^, CD8^+^ and γδ T cells (j) were analyzed by flow cytometry (n = 4 mice per group). k‐m, Vγ9Vδ2/αβ TILs combinatorial therapy effectively inhibits syngeneic CC tumor growth in a patient‐derived xenograft (PDX) model. k) Tumor extracted from CC patient CAT039 was used for establishing a PDX tumor in NCG mice and αβ TIL expansion, while peripheral blood derived from the same patient was used for Vγ9Vδ2 T cell expansion. Three *i.v*. injections were performed for each group with indicated medium or cells. Ctl, control culture medium. n = 4 mice per group. TIL, tumor‐infiltrating αβ T lymphocytes (1 × 10^7^ cells per mouse). γδT‐wt pep or γδT‐mu pep, pVγ9Vδ2 T cell (1 × 10^7^ cells per mouse) loaded with wildtype peptide pools or mutant peptide pools (neoantigens). γδT‐wt pep + TIL or γδT‐mu pep + TIL, 5 × 10^6^ pVγ9Vδ2 T cells + 5 × 10^6^ TIL per mouse. Tumor volume l) and mice survival m) were analyzed (n = 4 mice per group). Error bars represent mean with SD. *p* values were calculated using two‐tailed Student's *t* test. Survival curves were calculated with Log‐rank (Mantel‐Cox) test. *p < 0.05; **p < 0.01. NS, Not Significant.

We next examined if pVγ9Vδ2 T cells could be combined with αβ T cells to treat CC in vivo. To this end, a SiHa cell line‐derived xenograft (SiHa‐CDX) model was used. As demonstrated in **Figure** **5**a–c, SiHa‐CDX mice were adoptively transferred with pVγ9Vδ2 T cells (1st i.v.) followed by the 2nd *i.v*. injection of αβ T cell (were prepared as detailed in Figure S5b, Supporting Information) and pVγ9Vδ2 T cell mixtures either in the absence of CCLs loading (γδ/Mix‐unload group), or in the presence of CCLs loading (γδ/Mix‐load group). Compared to the control group (medium injected at both injections), the γδ/Mix‐unload group showed a delayed tumor growth. Interestingly, the γδ/Mix‐load group showed significantly delayed tumor growth and improved mice survival when compared to the control and γδ/Mix‐unload groups (Figure 5a–c). Similar results were obtained in the HeLa‐CDX model (Figure 5d–f), with γδ/Mix‐load group showing the best anti‐CC efficiency, suggesting the synergistic effect of pVγ9Vδ2 and αβ T cells (Figure 5a–f). Of note, there were no significant histological changes in major organs of all the recipient mice, as determined by H&E staining (Figure S7a, Supporting Information), indicating that mice are overall tolerant to our combinatorial T cell therapy.

Furthermore, tumor‐infiltrating immune cells were analyzed at day 10 after the second injection in SiHa‐CDX mice (Figure 5g–j). Consistent with previous results, inhibited tumor growth was observed in both groups with combinatorial therapy, especially in the γδ/Mix‐load group (Figure S7b,c, Supporting Information), accompanied by more post‐killing cavities formed in tumors (Figure 5g), and higher intensities of HLA‐DR, hCD4 and hCD107a staining (Figure 5h), as revealed by H&E and immunofluorescence microscopy, respectively. We next confirmed these findings using flow cytometry. As expected, hCD45^+^ human cells were undetectable in the tumor, blood, and spleen from control mice. In contrast, these cells were clearly observed in the tumor, and to a lesser extent, in blood and spleen from both groups with combinatorial therapy. Importantly, hCD45^+^ human cell infiltration was more frequently presented in the γδ/Mix‐load group compared to the γδ/Mix‐unload group (Figure 5i; Figure S7d,e, Supporting Information). With regard to T cell populations, the two treatment groups were comparable in CD8^+^ T cell infiltration, whereas the γδ/Mix‐load tumors tended to have more CD4^+^ T cells and less γδ T cells than the γδ/Mix‐unload tumors. More interestingly, tumor‐infiltrating T cells from the γδ/Mix‐load group exhibited a more differentiated phenotype, as indicated by more effector memory (T_EM_) and less central memory (T_CM_) cells in CD4^+^ T compartment, less naive (T_N_) cells in CD8^+^ T compartment, and more CD45RA^+^ T_EM_ (T_EMRA_) in γδ T compartment, than those from the γδ/Mix‐unload group (Figure S7f, Supporting Information), suggesting that T cells from the γδ/Mix‐load group might be more prone to differentiate into immediate killers. This notion was supported by intracellular staining of T cells, which revealed the higher frequencies of CD4^+^IFN‐γ^+^, CD4^+^TNF‐α^+^, and CD8^+^TNF‐α^+^ T cells in the γδ/Mix‐load group than the γδ/Mix‐unload group (Figure 5j; Figure S7g, Supporting Information).

To eliminate the possible alloreactivity of adoptively transferred T cells toward CC cell lines in the CDX models, in which effector cells and target cells are only partially HLA‐matched, we utilized PDX model to circumvent this problem. In this regard, NCG mice were divided into four groups upon implanted PDX tumors (from CC patient CAT039 described in Figure 4g) were palpable. Tumor‐bearing mice subsequently treated by medium only was served as the negative control, while those adoptively transferred with autologous αβ TILs were designated as the αβ TIL monotherapy group, and mice received autologous αβ TILs together with pVγ9Vδ2 T cells loaded with control antigens (i.e., wildtype peptide pools) or neoantigens (i.e., mutant peptide pools, Figure 4g) were designated as the two combinatorial therapy groups, respectively (Figure 5k). Compared with negative control, significantly delayed tumor growth and prolonged mouse survival were observed in all the treatment groups. Importantly, when αβ TILs and neoantigen‐loaded pVγ9Vδ2 T cells were used for combinatorial therapy, the therapeutic efficacy was higher than the other two treatment groups (Figure 5l,m). The anti‐CC activity observed in the three treatment groups is clearly resulted from adoptively transferred T cells, which is supported by the infiltration and activation of CD4^+^ and CD8^+^ T cells in the αβ TIL monotherapy group, and the presence of activated Vδ2, CD4^+^, and CD8^+^ T cells in the two combinatorial treatment groups (Figure S7h, Supporting Information), as determined by immunofluorescence staining of TCR Vδ2, CD4, CD8, and CD107a.

Taken together, our in vitro and in vivo findings suggest that combinatorial therapies with Vγ9Vδ2 and αβ T cell, especially when loaded with tumor‐specific antigens, are effective in treating CC.

### 2.6 Chemotherapy Potentiates γδ/αβ T Cell Combinatorial Immunotherapy in the Treatment of CC

**Figure 6:**
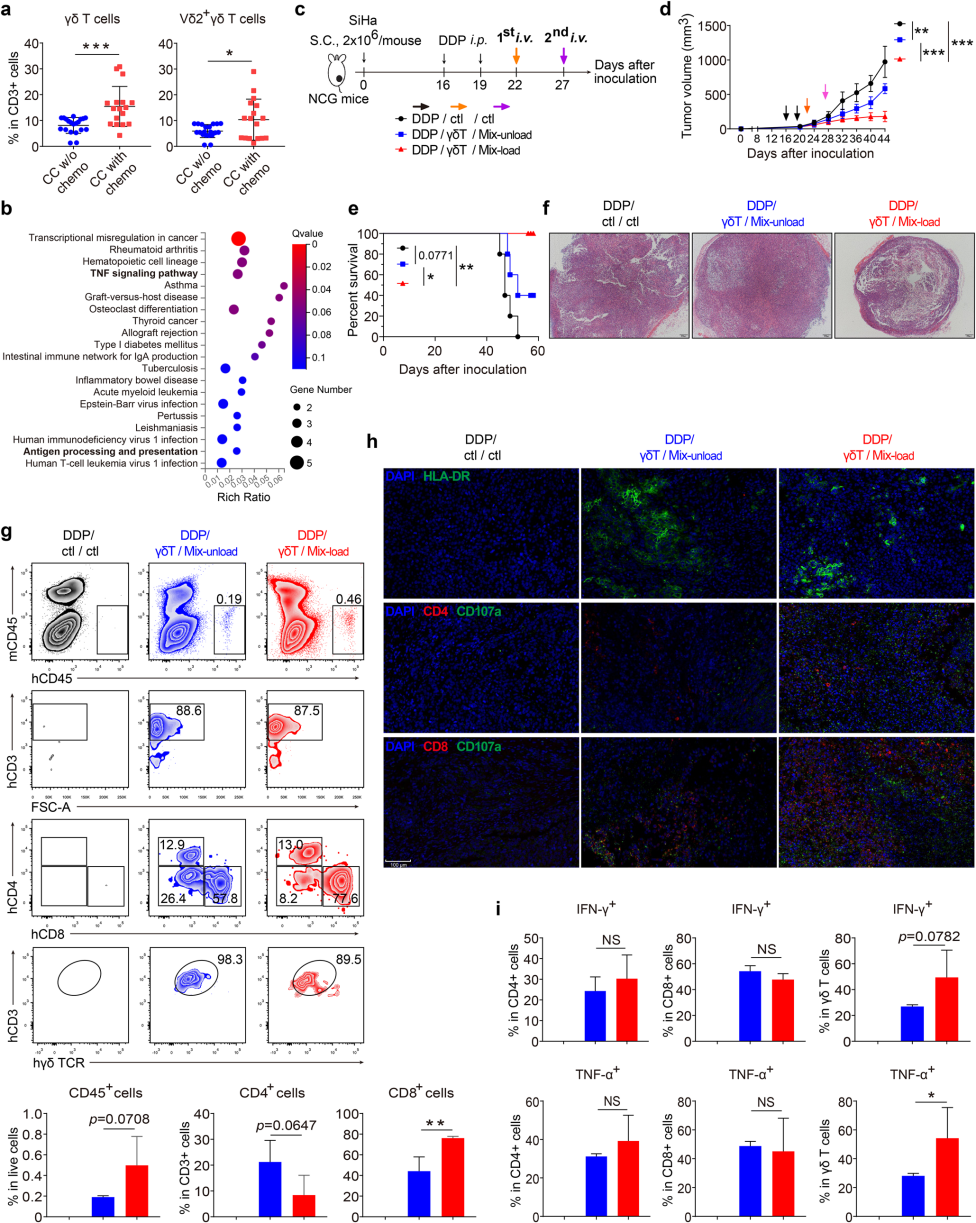
Vγ9Vδ2/αβ T cell combinatorial strategy plays a synergistic effect with chemotherapy in the treatment of CC. a) The proportion of peripheral total γδ T cell and Vδ2 T cell in CC patients with or without neoadjuvant chemotherapy was determined by flow cytometry. Chemo, chemotherapy. w/o, without. CC w/o chemo, n = 20. CC with chemo, n = 17. b) KEGG enrichment analysis of the up‐regulated genes in pVγ9Vδ2 T cells from CC patients with chemotherapy compared with those without chemotherapy. n = 2 per group. c‐e) γδ/αβ T cell combinatorial immunotherapy synergizes with DDP chemotherapy in a SiHa‐CDX model. c, Two *i.p*. injections with DDP (day 16 and 19 after SiHa cell inoculation) and two *i.v*. injections with indicated medium or cells were performed for three different treatment regimens. *i.p*., intraperitoneally. DDP, cis‐platin, 5 mg kg^−1^ body weight per mouse per day. γδT, Vγ9Vδ2 T cell (1 × 10^7^ cells per mouse). Mix‐unload and Mix‐load, 1 × 10^7^ cell mixtures that were described in Figure 5a. Tumor volume (d) and mice survival (e) were analyzed (n = 5 mice per group). The statistics in the tumor volume were calculated with the day 44 data. f‐i) In another set of experiments similar to c‐e, tumor‐infiltrating cells were determined at day 37 after SiHa cell inoculation (n = 4 mice per group). f) HE staining of tumor sections was analyzed. g, Single cell suspensions of tumor tissues were prepared, tumor infiltration of CD45^+^ cells, CD4^+^, CD8^+^ and γδ T cells were analyzed by flow cytometry. h, Activated T cells (HLA‐DR^+^), CD4^+^ T cell, CD8^+^ T cell and CD107a^+^ cells in the tumor tissues were analyzed by fluorescence microscopy. Scale bar: 100 µm. i, Single cell suspensions of tumor tissues were prepared, IFN‐γ‐ and TNF‐α‐producing CD4^+^, CD8^+^ and γδ T cells in tumor tissues were analyzed by flow cytometry. Error bars represent mean with SD. *p* values were calculated using two‐tailed Student's *t* test. Survival curves were calculated with Log‐rank (Mantel‐Cox) test. *p < 0.05; **p < 0.01; ***p < 0.001. NS, Not Significant.

Chemotherapy is a standard first‐line treatment regimen for CC, we therefore asked whether our combinatorial therapy can be applied after chemotherapy. It has been reported that certain chemotherapeutics could boost antitumor immunity directly through sensitizing cancer cells to T cell killing,^[^ ^45^, ^46^ ^]^ or indirectly by enhancing antigen presentation and counteracting immunosuppressive mechanisms.^[^ ^46^, ^47^ ^]^ In this study, higher expression levels of HLA‐DR, CXCR3 and PD‐1 on tumor‐infiltrating Vγ9Vδ2 T cells were observed in CC patients with neoadjuvant chemotherapy than those without (Figure S8a, Supporting Information). The effect of chemotherapy on γδ T cells in CC seems to be systemic, since the proportion of total γδ T, Vδ1, and Vδ2 T cells as well as the expression levels of HLA‐DR, CXCR3 and PD‐1 on the surface of peripheral Vδ2 T cell of patients with neoadjuvant chemotherapy, were higher than those of patients without chemotherapy (**Figure** **6**a; Figure S8b,c, Supporting Information). Moreover, transcriptomics analyses showed that compared to patients without chemotherapy, the up‐regulated genes in pVγ9Vδ2 T cell from patients with chemotherapy were mainly enriched in TNF signaling as well as antigen processing and presentation pathways (Figure 6b). Correspondingly, the transcriptional levels of some antitumor‐related cytokines, chemokines and antigen cross‐presentation molecules (purple circles labeled) were also higher in Vγ9Vδ2 T cells derived from patients with chemotherapy than those without (Figure S8d, Supporting Information).

Based on these results, we asked whether our γδ/αβ T cell immunotherapy could exert a synergistic effect with chemotherapy in treating CC. To test this premise, γδ/αβ T cell immunotherapies described in Figure 5 were applied after cDDP treatment in SiHa‐CDX mice (Figure 6c–e). Compared with cDDP treatment alone, cDDP followed by γδ/αβ T immunotherapies significantly inhibited tumor growth and prolonged mouse survival, especially when cells were loaded with tumor antigen (γδ/Mix‐load), which showed the best antitumor effect (Figure 6d,e). We next repeated the above experiments with the purpose of examining tumor‐infiltrating cells, similar results were obtained, with smaller and hollowed tumors, and a tendency of more tumor‐infiltrating immune cells in mice treated with cDDP followed by γδ/αβ T cell immunotherapies (Figure 6f,g; Figure S8e,f, Supporting Information). Interestingly, combination of cDDP and immunotherapy appeared to result in more infiltration of total leukocytes and CD8^+^ T cells in tumors than immunotherapy alone (Figure 5i; Figure 6g; Figure S8g–i, Supporting Information). More importantly, compared with those without antigen loading (γδ/Mix‐unload), when loaded with antigens (γδ/Mix‐load), γδ/αβ T cell immunotherapy applied after chemotherapy resulted in significantly more CD8^+^ T cell infiltration (Figure 6g). These results were echoed in the immunofluorescence assays, which showed a very impressive infiltration and activation of CD8^+^ T cells in tumor tissues of mice with chemotherapy and γδ/Mix‐load sequential treatments (Figure 6h). In addition, although tumor‐infiltrating CD4^+^, CD8^+^, and γδ T cells all showed high expression of IFN‐γ and TNF‐α in both groups with immunotherapy, the frequency of TNF‐α^+^ γδ T cells was markedly higher when transferred cells were loaded with antigen (Figure 6i; Figure S8j, Supporting Information). Of note, no significant side effects were observed in major organs of all the recipient mice in above experiments (Figure S8k, Supporting Information).

Taken together, these results suggest that Vγ9Vδ2/αβ T cell combinatorial immunotherapy could play a synergistic effect with chemotherapy in the treatment of CC.

### 2.7 Vγ9Vδ2 T Cell Counteracts BTN3A1‐Mediated Inhibition of αβ T Cell Response by Competitively Binding to BTN3A1

Given the highest therapeutic efficacy of combinatorial immunotherapy with neoantigen‐loaded pVγ9Vδ2 T cells and αβ TIL among all groups compared (Figure 5k–m), and the strong antigen‐presenting capacity of our expanded Vγ9Vδ2 T cells (Figure 4), it is reasonable to attribute the synergistic anti‐CC effect of Vγ9Vδ2 T cells and αβ TILs to Vγ9Vδ2 T cell‐mediated antigen presentation. Nevertheless, pVγ9Vδ2/αβ TIL combinatorial immunotherapy already outcompeted αβ TIL monotherapy even without the presence of tumor neoantigen (Figure 5l,m), suggesting that pVγ9Vδ2 T cells might also function through antigen presentation‐independent mechanisms such as direct killing. However, irradiated Vγ9Vδ2 T cells, which clearly lack the killing ability (Figure S9a, Supporting Information), were still capable of promoting the tumoricidal capacity of DMF5 TCR‐T cells even without the presence of Mart‐1_27‐35_ peptides (Figure S9b,c, Supporting Information), indicating that pVγ9Vδ2 T cells might promote αβ T cell activation through a novel mechanism independent of antigen presentation and direct killing. Interestingly, the synergistic effect can be reversed by the addition of anti‐TCR γδ neutralizing antibody (Figure S9b, Supporting Information), suggesting that TCR γδ is required for αβ T cell‐promoting effect of irradiated Vγ9Vδ2 T cells.

**Figure 7:**
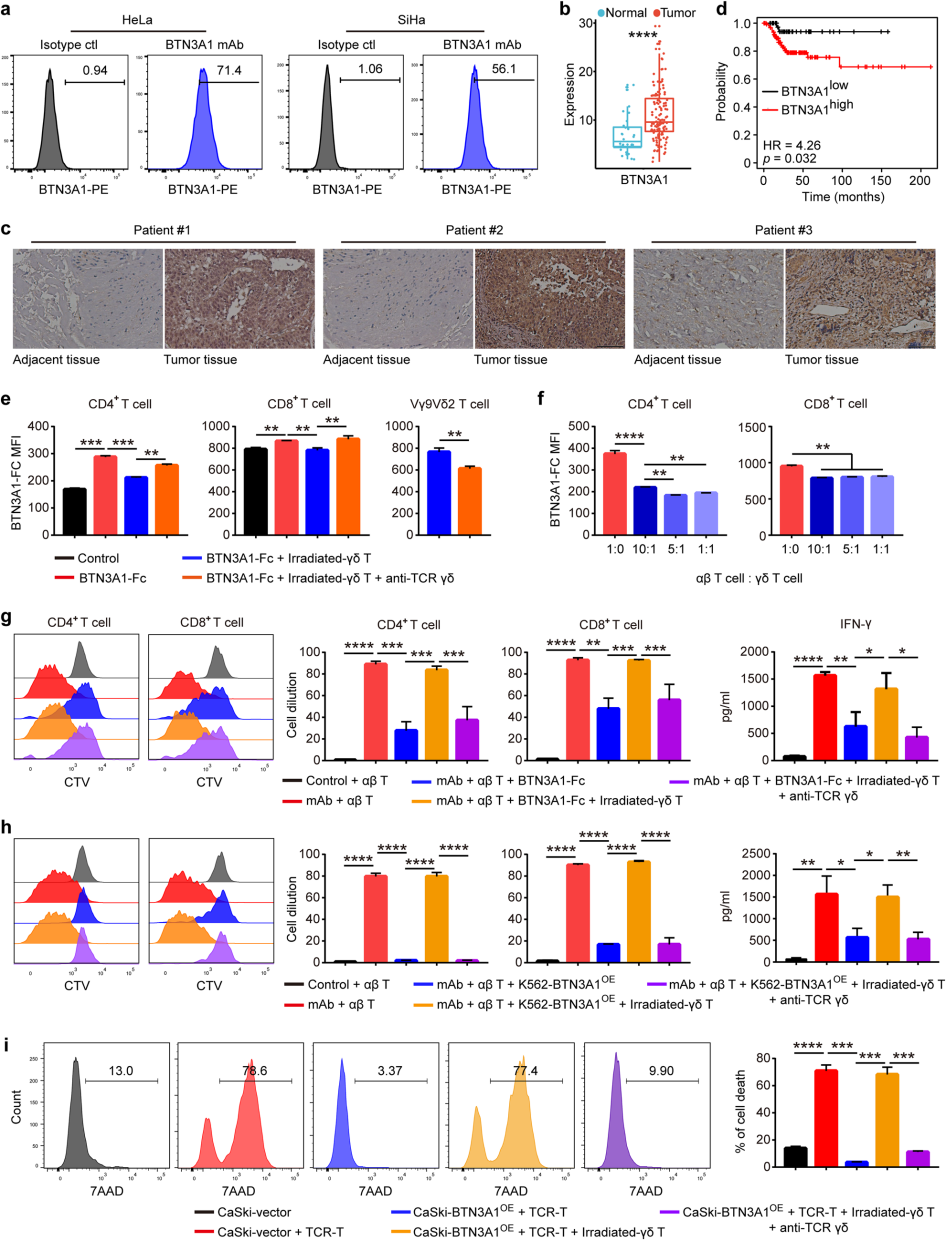
Vγ9Vδ2 T cell restores BTN3A1‐mediated inhibition of αβ T cell response by competitively binding to BTN3A1. a) BTN3A1 expression levels on CC cell lines HeLa and SiHa were determined by flow cytometry. b) BTN3A1 expression level in normal and tumor tissues of CC was determined by RNA sequencing (normal, n = 32; tumor, n = 127). c) Representative images showing BTN3A1 expression level in tumor and adjacent tissues of CC determined by immunohistochemistry. Scale bar: 200 µm. d) The correlation of BTN3A1 expression level with the prognosis of CC patients (Relapse Free Survival, RFS) from TCGA dataset was analyzed (n = 306, graphed in Kaplan‐Meier Plotter database, Log‐rank test was used). e) The binding of BTN3A1‐Fc fusion protein to the surface of CD4^+^ and CD8^+^ T cells in the presence or absence of irradiated pVγ9Vδ2 T cell ± TCR γδ‐blocking antibody was determined by flow cytometry (n = 3 per group). IgG‐FC (10702‐HNAH, Sino Biologic) was used as control. f) The binding of BTN3A1‐Fc fusion protein to the surface of CD4^+^ and CD8^+^ T cells in the presence or absence of the indicated ratios of irradiated pVγ9Vδ2 T cell was determined by flow cytometry (n = 3 per group). g, h) Naïve αβ T cells were cultured and stimulated by CD3 and CD28 mAbs. BTN3A1‐Fc (g) or K562‐BTN3A1^OE^ (h), irradiated pVγ9Vδ2 T cell and TCR γδ‐blocking antibody were added to the cultures in the indicated conditions. CD4^+^ and CD8^+^ T cells proliferation and the concentration of IFN‐γ in the culture supernatants were determined by flow cytometry and ELISA respectively (n = 3 per group). i) CaSki‐vector or CaSki‐BTN3A1^OE^ cells were cultured with Mart‐1_27‐35_ specific TCR‐T cells for 12 h. Irradiated pVγ9Vδ2 T cell and TCR γδ‐blocking antibody were added to the cultures in the indicated conditions. The percentage of CaSki cell death was determined by flow cytometry (n = 3 per group). Error bars represent mean with SD. *p* values were calculated using two‐tailed Student's *t* test. *p < 0.05; **p < 0.01; ***p < 0.001; ****p < 0.0001. OE, overexpression. mAb, anti‐CD3 and anti‐CD28 monoclonal antibodies.

As a key ligand of TCR Vγ9Vδ2, BTN3A1 has been well‐established for its critical role in inducing Vγ9Vδ2 T cell activation.^[^ ^26^ ^]^ However, BTN3A1‐mediated pVγ9Vδ2 T cell activation is unlikely responsible for the synergistic effect described in the above coculture system due to the use of irradiated pVγ9Vδ2 T cells (Figure S9b,d, Supporting Information). Interestingly, knocking out BTN3A1 in CaSki cells (CaSki‐BTN3A1^KO^) by CRISPR‐Cas9 eliminated the αβ T cell‐promoting effect of pVγ9Vδ2 T cells, which was not affected by blocking TCR γδ (Figure S9c,d, Supporting Information). These results suggest that BTN3A1‐TCR γδ interaction is necessary for pVγ9Vδ2 T cells to exert synergistic function with αβ T cells through mechanisms independent of activating Vγ9Vδ2 T cells. A recent study reported that BTN3A1 could inhibit αβ T cell activation through binding to CD45.^[^ ^48^ ^]^ Indeed, a variety of CC cell lines including HeLa, SiHa, C33A, ME‐180, and MS751 expressed high level of BTN3A1 (**Figure** **7**a; Figure S9e,f, Supporting Information). Furthermore, BTN3A1 was highly expressed in tumor tissues of CC, as revealed by transcriptomics and immunohistochemistry analyses (Figure 7b,c). Importantly, the TCGA data showed that the expression level of BTN3A1 in CC tissues was negatively correlated with the prognosis of patients^[^ ^49^ ^]^ (Figure 7d). Therefore, we hypothesized that BTN3A1 on the surface of CC cells may inhibit the antitumor effects of αβ T cells as previously reported,^[^ ^48^, ^50^, ^51^ ^]^ while Vγ9Vδ2 T cells likely relieve this inhibition by competitively binding to BTN3A1 through TCR γδ. Indeed, BTN3A1‐Fc fusion protein but not control protein could bind to the surface of CD4^+^ and CD8^+^ T cells, whereas this binding was down‐regulated by the addition of irradiated pVγ9Vδ2 T cell to the culture system, accompanied by increased binding of BTN3A1 to pVγ9Vδ2 T cells (Figure 7e). In contrast, the above effects were abolished when the TCR γδ‐blocking antibody was added simultaneously. Remarkably, pVγ9Vδ2 T cells were still able to inhibit the binding of BTN3A1 to the surface of CD4^+^ and CD8^+^ T cells even when the pVγ9Vδ2 T cell to αβ T cell ratio was as low as 1:10 (Figure 7f). These results suggest that pVγ9Vδ2 T cells could bind to BTN3A1 through their TCR with greater affinity than αβ T cells, thereby competitively inhibiting the interaction between BTN3A1 and αβ T cells.

To further explore whether Vγ9Vδ2 T cell can reverse BTN3A1‐mediated suppression of αβ T cell response, we added BTN3A1‐Fc fusion protein to an in vitro system of αβ T cell proliferation with or without the addition of irradiated pVγ9Vδ2 T cells. Consistent with the results of the binding assay, BTN3A1 inhibited the capacity of CD4^+^ and CD8^+^ T cells activated by anti‐CD3/CD28 antibodies to proliferate and secrete IFN‐γ, whereas pVγ9Vδ2 T cells can reverse this inhibitory effect of BTN3A1 in a TCR γδ‐dependent manner (Figure 7g; Figure S9g, Supporting Information). To better simulate the in vivo effects of BTN3A1, which is expressed on the cell membrane of tumor cells,^[^ ^52^ ^]^ we transfected BTN3A1 into K562 cells, which showed very low baseline BTN3A1 expression, to establish BTN3A1‐overexpressed K562 cells (K562‐BTN3A1^OE^) (Figure S9i, Supporting Information), and used it (Mitomycin C pretreated) to replace BTN3A1‐Fc fusion protein used in above experiments. Consistent with that obtained with BTN3A1‐Fc, K562‐BTN3A1^OE^ was also effective in inhibiting the proliferation and differentiation of CD4^+^ and CD8^+^ T cells, which can be similarly reversed by the addition of irradiated pVγ9Vδ2 T cells. (Figure 7g,h; Figure S9g,h, Supporting Information). Furthermore, DMF5 TCR‐T cell could effectively kill Mart‐1_27‐35_ peptide‐pulsed HLA‐A2^+^ CaSki cells, which express a low level of BTN3A1 (Figure S9e, Supporting Information), but this killing effect was markedly reduced when CaSki cells were overexpressed with BTN3A1 (Figure 7i; Figure S9d, Supporting Information). Importantly, the addition of irradiated pVγ9Vδ2 T cells could restore the killing effect of TCR‐T cells by counteracting BTN3A1 in a TCR γδ‐dependent manner (Figure 7i).

Taken together, these results suggest that pVγ9Vδ2 T cell could counteract BTN3A1‐mediated suppression of antitumor αβ T cell response by competitively binding to BTN3A1, and this mechanism may be partially responsible for the effects of these cells in promoting the antitumor function of αβ T cells or TILs as described in Figure 5k–m.

### 2.8 Activated T Cell Induces the Expression of BTN3A1 in CC Cells in an IFN‐γ‐Dependent Manner

**Figure 8:**
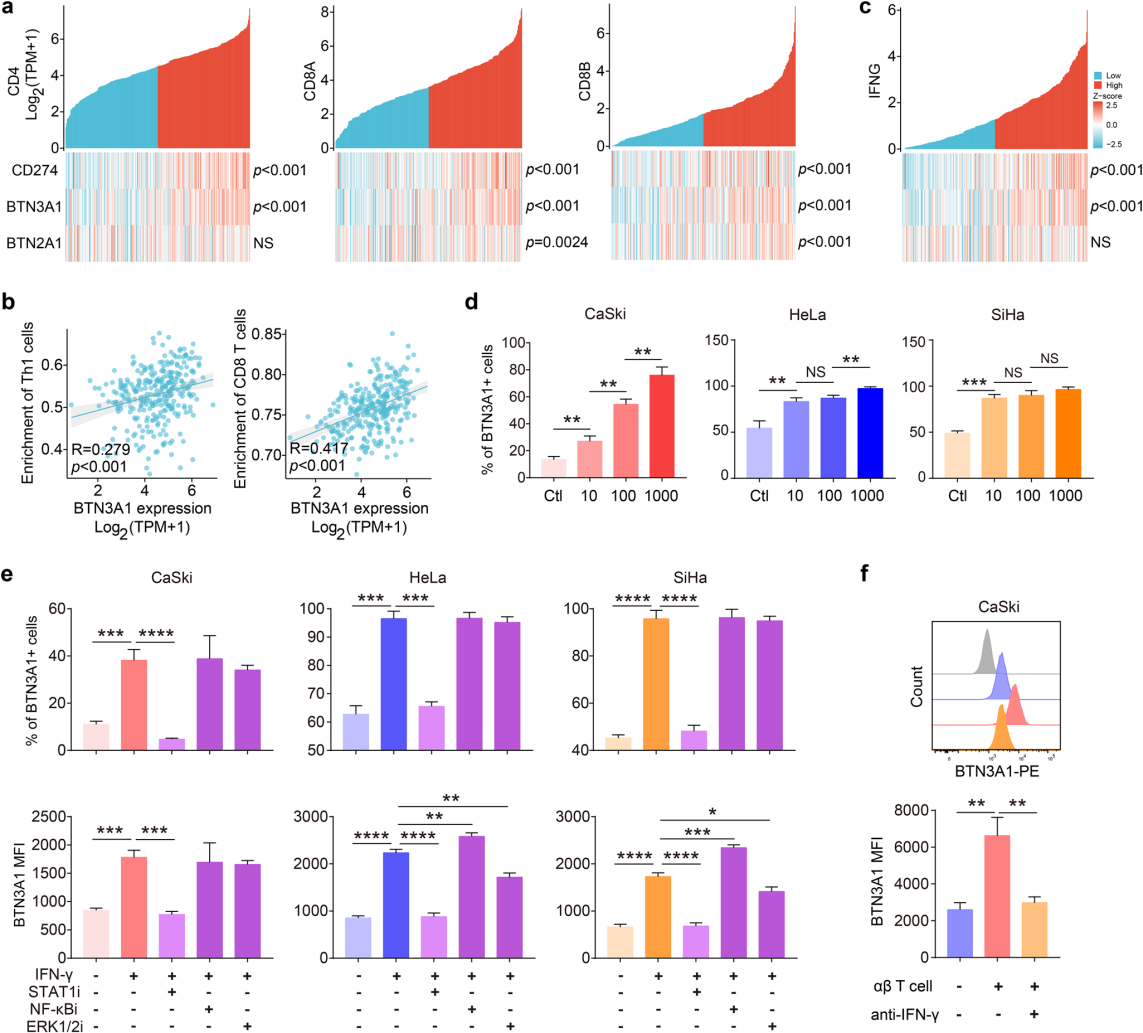
IFN‐γ derived from activated αβ T cell induces the expression of BTN3A1 in CC cells. a) The correlation of *CD274*, *BTN3A1* and *BTN2A1* expression levels with *CD4*, *CD8A* and *CD8B* expression levels in CC from TCGA dataset were analyzed (n = 306). b) The correlation of *BTN3A1* expression level with the abundance of tumor‐infiltrating Th1 and CD8^+^ T cells in CC from TCGA dataset was analyzed (n = 306). c) The correlation of *CD274*, *BTN3A1*, and *BTN2A1* expression levels with *IFNG* expression level in CC from TCGA dataset were analyzed (n = 306). d) CaSki, HeLa and SiHa cells were cultured and stimulated by the indicated concentrations of IFN‐γ (ng mL^−1^) for 24 h. The percentage of BTN3A1^+^ cells was determined by flow cytometry (n = 3 per group). e) CaSki, HeLa and SiHa cells were cultured and stimulated by the indicated concentrations of IFN‐γ (ng mL^−1^), in the absence or presence of STAT1, NF‐κB, or ERK1/2 inhibitor, for 24 h. The percentage of BTN3A1^+^ cells and BTN3A1 intensity were determined by flow cytometry (n = 3 per group). f) CaSki cell was cultured with activated αβ T cells, in the absence or presence of IFN‐γ blocking antibody, for 48 h. The intensity of BTN3A1 on CaSki cells was determined by flow cytometry (n = 3 per group). Error bars represent mean with SD. *p* values were calculated using Spearman statistics and two‐tailed Student's *t* test. *p < 0.05; **p < 0.01; ***p < 0.001; ****p < 0.0001. NS, Not Significant.

Although the inhibitory effect of BTN3A1 on the activation and killing capacities of αβ T cell has been reported,^[^ ^48^, ^50^, ^51^ ^]^ the regulatory mechanism of BTN3A1 expression in tumor cells remains unclear. By analyzing the database,^[^ ^53^ ^]^ we found that *BTN3A1* was positively correlated and co‐expressed with an array of previously described IFN‐γ‐induced molecules^[^ ^54^, ^55^ ^]^ in CC tissues, but not in tumor adjacent tissues or normal cervical tissues (Table S3, Supporting Information). Furthermore, the TCGA data showed that although the expression levels of *BTN3A1, BTN2A1*, and *PD‐L1* were all positively correlated with *CD8A* and *CD8B* in CC, those of *BTN3A1* and *PD‐L1*, but not *BTN2A1* were correlated with *CD4* and *IFNG*, as well as the abundance of CD4^+^ Th1 and CD8^+^ T cells in CC (**Figure** **8**a–c). It has been well‐established that IFN‐γ secreted by activated T cells could induce tumor cells to express PD‐L1.^[^ ^56^ ^]^ Therefore, we speculated that the expression of BTN3A1 in CC cells might be regulated through similar mechanism. Consistent with this premise, IFN‐γ stimulation could increase the expression of BTN3A1 in various CC cells in a dose‐dependent manner (Figure 8d; Figure S10a, Supporting Information). In addition, we found that IFN‐γ‐induced BTN3A1 expression in CC cells was dependent on JAK‐STAT1 signaling pathway, which is reminiscent of the mechanism of IFN‐γ‐induced PD‐L1 expression in tumor cells^[^ ^57^ ^]^ (Figure 8e; Figure S10b, Supporting Information). More interestingly, activated T cells could promote the expression of BTN3A1 on the surface of CC cells, while the addition of IFN‐γ neutralizing antibody to the co‐culture system significantly inhibited the expression of BTN3A1 (Figure 8f). Thus, these results suggest that activated T cells in CC can induce the expression of BTN3A1 in tumor cells in an IFN‐γ‐dependent manner.

## 3 Discussion

Patients with advanced/metastatic CC are suffered from lacking effective treatments, and therefore have a poor prognosis. In this study, we report a synergistic effect of autologous pVγ9Vδ2 T cell and αβ T cell in immunotherapy of CC. By using both in vitro and in vivo models, we found that expanded pVγ9Vδ2 T cell is not only a strong tumor killer, but also capable of potentiating the antitumor effect of αβ T cell by antigen presentation and counteracting BTN3A1‐mediated αβ T cell inhibition.

The antitumor effect of γδ T cell has been recognized dating back to 1980s shortly after this cell was discovered,^[^ ^58^, ^59^, ^60^, ^61^, ^62^ ^]^ and has received more attentions since γδ T cell was reported to be the most significant signature associated with the favorable prognosis across a variety of cancers.^[^ ^25^, ^39^, ^63^, ^64^, ^65^ ^]^ However, to date, the role of γδ T cell in CC is still not well‐understood. By analyzing the database and our clinical data (Figure 1; Figure S1, Supporting Information), we were able to show that the frequency of γδ TILs was closely correlated with the better prognosis of CC as well as the infiltration and activation of various antitumor immune cells. Interestingly, Vγ9Vδ2 T cell, a γδ T cell subset predominantly found in the periphery,^[^ ^24^, ^36^ ^]^ was detected in CC tissue at a frequency comparable to and even higher in some patients than Vδ1 T cell (Figure 1j), the well‐known tissue‐resident γδ T cell subset.^[^ ^36^ ^]^ Importantly, the frequency of Vγ9Vδ2 TILs (Figure 1l) but not Vδ1 TILs, was found to be significantly associated with αβ T cell abundance, indicating the connection between Vγ9Vδ2 T cell and αβ T cell in CC. Indeed, Vγ9Vδ2 T cell has been reported to present and cross‐present antigen to αβ T cell with a high efficiency comparable to DC.^[^ ^27^, ^28^, ^29^ ^]^ In this study, we verified the strong antigen‐processing and ‐presenting abilities of pVγ9Vδ2 T cell toward model antigens (i.e., DQ‐OVA and Mart‐1). More importantly, pVγ9Vδ2 T cell is also able to present and cross‐present CC‐derived antigens (i.e., CCLs and neoantigens) to CD4^+^ and CD8^+^ αβ T cells in both in vitro experiments (Figure 4), and in vivo models with CDX and PDX (Figure 5). Therefore, the synergistic anti‐CC effect of autologous pVγ9Vδ2 T cells and αβ T cells observed in our animal models can be attributable to the direct tumor‐killing and antigen presentation capacities of pVγ9Vδ2 T cells. In this scenario, through killing of CC cells, pVγ9Vδ2 T cells may not only result in tumor regression directly (the first hit), but also indirectly by causing the release of tumor antigens, which are subsequently processed and presented to tumor‐specific αβ T cells, and thereby confer “the second hit”. The latter mechanism is dependent on the cognate interaction between these two cell types. Thus, it is not surprising that pVγ9Vδ2/αβ T cell combinatorial therapies are more effective when CC antigens are loaded (Figure 5), in which more cognate interactions are expected.

In addition to direct tumor‐killing and antigen presentation, we also uncover another synergistic mechanism of pVγ9Vδ2/αβ T cell combinatorial therapy, through which pVγ9Vδ2 T cells reverse BTN3A1‐mediated αβ T cell inhibition by competitively binding to BTN3A1 expressed by CC cells (Figure 7). BTN3A1 is widely expressed in a variety of cells including immune cells and tumor cells.^[^ ^66^, ^67^ ^]^ BTN3A1 expressed by APC is crucial for pAg‐induced activation of Vγ9Vδ2 T cells by forming a heterodimer with BTN2A1.^[^ ^68^, ^69^ ^]^ In this study, we found that BTN3A1 is also expressed on various CC cells. However, BTN3A1‐mediated pVγ9Vδ2 T cell activation is dispensable, at least in vitro, for the synergistic effect of pVγ9Vδ2 T cell and αβ T cell, since irradiated pVγ9Vδ2 T cell, which clearly cannot respond to any stimulation, could also synergize with αβ T cell in killing BTN3A1‐overexpressed but not BTN3A1‐deficient CC cells in a TCR γδ‐dependent manner. These results, together with a recent finding that BTN3A1 can significantly inhibit the antitumor effect of αβ T cell by abrogating TCR activation in ovarian cancer,^[^ ^48^ ^]^ are suggestive of BTN3A1 as a novel checkpoint molecule and an alternative function of Vγ9Vδ2 T cell as a “cellular ICI”.

Except for CC cell lines, CC tissues also express high level of BTN3A1, but the mechanism by which BTN3A1 expression is regulated in CC cells remains unknown. Earlier studies reported that IFN‐γ or TNF‐α could promote the expression of BTN3 molecules in endothelial cells.^[^ ^70^ ^]^ Recent study also showed that IFN‐γ can promote Vγ9Vδ2 T cell activation by inducing monocytes to express BTN3A1.^[^ ^71^ ^]^ This study provides several lines of evidence supporting that BTN3A1 expression is primarily regulated by IFN‐γ in CC cells (Figure 8). First, the expression of BTN3A1 in CC tissues was significantly correlated and co‐expressed with IFN‐γ and a variety of IFN‐γ inducible molecules.^[^ ^54^, ^55^ ^]^ Second, IFN‐γ could induce the expression of BTN3A1 in CC cells in a JAK1‐ and STAT1‐dependent manner. Third, activated αβ T cells could promote the expression of BTN3A1 on the surface of CC cells, while blocking IFN‐γ with a neutralizing antibody reversed αβ T cell‐induced expression of BTN3A1. More interestingly, the infiltration levels of CD4^+^ and CD8^+^ T cells and the expression level of IFN‐γ in CC tissues were correlated with the expression of BTN3A1 and PD‐L1, but not BTN2A1. Thus, the expression pattern, regulatory mechanism, and αβ T cell inhibiting effect of BTN3A1 make it highly similar to PD‐L1,^[^ ^57^ ^]^ further elaborating the notion that BTN3A1 expressed by tumor cells may be another immune checkpoint molecule rather than merely activate Vγ9Vδ2 T cells with BTN2A1 in CC.

Although chemotherapy has been reported to be capable of improving the tumoricidal activity of T cells including γδ T cells in a variety of cancers ranging from colon cancer, ovarian cancer, glioblastoma, and chronic lymphocytic leukemia,^[^ ^45^, ^46^, ^72^, ^73^, ^74^ ^]^ this study is the first to reveal the boosting effect of chemotherapy on pVγ9Vδ2/αβ T cell combinatorial therapies in CC. Chemotherapy may potentiate antitumor response through increasing the sensitivity of tumor cells to T cell‐mediated killing,^[^ ^45^, ^46^ ^]^ and/or recruiting and activating tumoricidal T cells.^[^ ^75^, ^76^, ^77^ ^]^ In this study, we found that the abundance and antitumor potential of Vγ9Vδ2 T cells in peripheral and tumor tissues of CC patients experienced chemotherapy were higher than that of without chemotherapy experience. Importantly, elevated infiltration and increased activation of transferred T cells, especially CD8^+^ T cells, were observed in CC tissue of mice treated with pVγ9Vδ2/αβ T cells and chemotherapy than those with pVγ9Vδ2/αβ T cell therapy alone (Figure 6g,h). This effect was partially consistent with a previous report that chemotherapeutic drugs could promote the infiltration of CAR‐T cells in lung tumor through inducing the production of T cell‐recruiting chemokines.^[^ ^78^ ^]^ Nevertheless, the specific mechanism underlying the boosting effect of chemotherapy on pVγ9Vδ2/αβ T cell combinatorial therapies observed in our study needs to be further explored.

The expansion efficiency and purity of γδ T cells are critical factors influencing their application in tumor immunotherapy. High expansion efficiency ensures a sufficient number of γδ T cells for therapeutic use, which is essential given the typically low frequency of γδ T cells in peripheral blood. Previous studies have shown that the amplification efficiency of autologous pVγ9Vδ2 T cells in some patients with advanced cancer may be poor.^[^ ^37^ ^]^ Therefore, therapeutic strategies with allogeneic pVγ9Vδ2 T cells were explored alternatively.^[^ ^39^, ^74^, ^79^, ^80^, ^81^, ^82^, ^83^ ^]^ However, our results demonstrated that pVγ9Vδ2 T cells from advanced CC patients have similar amplification potential to that from HC (Figure 3a; Figure S3a, Supporting Information). How the functionality of peripheral Vγ9Vδ2 T cells is regulated in CC remains unclear. Nevertheless, CC‐derived pVγ9Vδ2 T cells exhibit higher antitumor potential than HC‐derived counterparts (Figure 2b–e). Importantly, after expansion, pVγ9Vδ2 T cells from CC and HC are highly similar in expression profiles at both transcriptional and protein levels (Figure 3b–d), indicating the functional convergence of pVγ9Vδ2 T cells from different sources after expansion. Therefore, from the manufacturing point of view, expanded autologous pVγ9Vδ2 T cells is a reasonable option for ACT therapy of CC. Of note, most of the functional experiments conducted in this study used pVγ9Vδ2 T cells expanded for 11 days instead of 14 days, with the classical protocol of IL‐2 plus zoledronate.^[^ ^38^, ^84^ ^]^ Here, pVγ9Vδ2 T cells can be expanded to several thousand folds in D11 cell products, which is sufficient for ACT. Importantly, compared to the D14 counterparts, D11 pVγ9Vδ2 T cells have higher expression of activation and effector molecules (e.g., CD86, HLA‐DR, NKG2D, perforin and granzyme B) and less inhibitory molecules (e.g., LAG3, TIM3, TIGIT and TOX), suggesting that shortened amplification protocol might not only expedite the manufacture process, but also preserve antitumor potential of pVγ9Vδ2 T cells (Figure 3; Figure S3, Supporting Information). Notably, the expansion of γδ T cells for clinical immunotherapy still faces significant challenges. On the one hand, there is a high demand for the number of cells required; on the other hand, contamination with other cell types must be minimized. Lower purity or suboptimal expansion conditions may lead to reduced anti‐tumor efficacy and an increased risk of off‐target effects. This highlights the need for stringent quality control during the preparation of γδ T cells for adoptive cell therapies. Several recent studies have reported that various modified protocols (e.g., with the addition of TGF‐β, vitamin‐C and rapamycin, etc)^[^ ^63^, ^85^, ^86^ ^]^ can enhance the amplification efficiency, purity, functionality and stemness of pVγ9Vδ2 T cells after 14‐day's expansion. Thus, future experiments are required to test these recipes and novel protocols using our 11‐day expansion regimen.

Of note, given the versatile antitumor capacity of Vγ9Vδ2 T cell,^[^ ^23^, ^24^, ^25^ ^]^ this cell could be potentially combined with other immunotherapeutic strategies other than ACT of αβ T cell, such as ICI and therapeutic vaccines, which have been often restrained by the lack of tumor‐specific T cells, impairment of antigen presentation, and emergence of additional checkpoint molecules, for treating CC patients.^[^ ^10^, ^21^, ^87^ ^]^ Thus, future investigations are encouraged to test these possibilities.

In summary, the findings of this study highlight a critical synergistic effect of autologous pVγ9Vδ2 T cells and αβ T cells that can be further boosted by chemotherapy for immunotherapy of CC and elucidate the potential underlying mechanisms. Our study provides the first proof‐of‐concept evidence for utilizing autologous Vγ9Vδ2/αβ T cell combinatorial therapy for treating CC patients. Future studies are anticipated to test these results in the clinical setting, and to explore the possibility of applying this strategy to a wide range of tumors.

## 4 Experimental Section

### Human Samples

The study was approved by the Medical Ethics Committee of the Tongji Medical College, Huazhong University of Science and Technology, Wuhan, China (Approval No. [2021] S034). Thirty‐seven CC patients participated in this study (Table S1, Supporting Information), including 20 CC patients without chemotherapy (peripheral blood and tumor tissues were all collected) and 17 CC patients with chemotherapy (peripheral blood and tumor tissues were collected in 9 cases, and peripheral blood was collected in the other 8 cases). HC‐derived peripheral blood samples were obtained from 10 subjects. Written informed consents were obtained from all participants before study entry.

### Cell Lines and Cell/Tissue Cultures

The CC cell lines SiHa, HeLa, C‐33A, and MS751, and the human umbilical vein endothelial cell line HUVEC were cultured in Dulbecco's Modified Eagle's Medium supplemented with 10% heat‐inactivated FBS (Gibco, Life Technologies) and 1% penicillin/streptomycin. The CC cell line CaSki and the human chronic myelogenous leukemia cell line K562 were cultured in RPMI 1640 medium supplemented with 10% FBS (Gibco, Life Technologies) and 1% penicillin/streptomycin. The CC cell line ME‐180 was cultured in modified McCoy's 5a medium supplemented with 10% heat‐inactivated FBS (Gibco, Life Technologies) and 1% penicillin/streptomycin. All these cell lines were maintained at 37 °C in a humidified atmosphere of 5% CO_2_, and tested bimonthly for mycoplasma and were mycoplasma‐free at the time of experiments. The CaSki‐BTN3A1^OE^ and K562‐BTN3A1^OE^ cell lines were established by stably transducing with a lentivirus vector encoding the hBTN3A1‐GFP. For in vitro experiments, expanded HC and CC patients‐derived γδ T cells, αβ TIL,^[^ ^88^ ^]^ and patient‐derived EBV‐LCL cells established from PBMC were generated as described previously,^[^ ^88^ ^]^ and were cultured in RPMI 1640 medium supplemented with 10% FBS (Gibco, Life Technologies) and 1% penicillin/streptomycin.

### Culture, In Vitro Expansion, and Transfection of Human Peripheral γδ T Cells

HC‐ and CC‐derived peripheral blood mononuclear cells (PBMC) were isolated using a Ficoll‐Paque‐based density gradient centrifugation protocol. PBMC were cultured in X‐Vivo medium supplemented with 10% heat‐inactivated FBS (Gibco, Life Technologies) at 2 × 10^6^ cells per well. To induce the expansion of Vγ9Vδ2 T cell, zoledronate (5 µM working concentration, Sigma) and recombinant human IL‐2 (1000 IU mL^−1^ working concentration, clinical grade, Beijing Sihuan Bio‐pharmaceutical Co., LTD) were added to the culture medium on day 0. After 4 days of stimulation, the culture system was replaced with a new medium containing 5 µM zoledronate and 1000 IU mL^−1^ rhIL‐2. On day 6 of culture, the culture system was replaced again with a medium containing 1000 IU mL^−1^ rhIL‐2. In subsequent cultures, the cells were counted daily and a new medium containing 1000 IU mL^−1^ of rhIL‐2 was added to keep the cell density in the range of 0.8–1.2 × 10^6^ mL^−1^. On the 8th, 11th, and 14th day of cultures, a certain number of cultured cells were taken to detect the characteristics of Vγ9Vδ2 T cell including purity, activation, differentiation, killing and antitumor potential. According to the counting results, the amplification ratio and efficiency of Vγ9Vδ2 T cells were calculated after different days of culture. For some experiments (RNA sequencing, in vitro killing assays, co‐culture experiments and in vivo adoptive transfer), γδ T cells were all obtained through magnetic sorting after expansion. Specifically, γδ T cells were first treated with a PE‐labeled Vδ2 antibody (Clone B6) and then sorted and purified using an anti‐PE magnetic bead sorting kit (130‐048‐801, Miltenyi Biotech). After sorting, the purity of Vγ9Vδ2 T cells was above 90%.

In order to construct Vγ9Vδ2 T cells that can present intracellular Mart‐1 antigenic peptide, DNA sequences encoding the Mart‐1 sequence (ATGTACACCACCGCCGAGGAGCTGGCCGGCATCGGAATCCTGACCGTGATCCTGGGCGTGTAA), signal sequence of LAMP1 (sig, ATGGCGGCCCCCGGCAGCGCCCGGCGACCCCTGCTGCTGCTACTGCTGTTGCTGCTGCTCGGT), and DC.LAMP sequence (TGCTCGTCTGACTACACAATTGTGCTTCCTGTGATTGGGGCCATCGTGGTTGGTCTCTGCCTTATGGGTATGGGTGTCTATAAAATCCGCCTAAGGTGTCAATCATCTGGATACCAGAGAATCTAA) were synthesized and cloned into a pcDNA3.1 based Mart‐1 vector (pcDNA3.1‐sig‐Mart‐1‐DC.LAMP vector). For electroporation of pcDNA3.1‐sig‐Mart‐1‐DC.LAMP vector, expanded autologous Vγ9Vδ2 T cell (1 × 10^6^‐5 × 10^6^) were resuspended in 100 µL Opti‐MEM (Life Technologies) and electroporated with 5 µg of vector using the electroporation cuvette and an electroporator (Lonza). Electroporated cells were transferred into a 2 ml complete medium, and cultured overnight at 37 °C, 5% CO_2_ for subsequent coculture assays. Transfection efficiency (determined by a control pcDNA3.1‐eGFP vector) was > 50%.

### Flow Cytometry

The human antibodies used for flow cytometry are as follows: CD45 (clone H130, BD), CD3 (clone SK7, BD), γδ TCR (clone B1, Biolegend), CD4 (clone RPA‐T4, BD), CD8 (clone SK1, BD), CD16 (clone 3G8, BD), CD40 (clone 5C3, BD), CD56 (clone B159, BD), CD86 (clone IT2.2, Biolegend), CD54 (clone HA58, BD), TCR Vδ1 (clone TS8.2, Invitrogen), TCR Vδ2 (clone REA771, Miltenyi Biotec), TCR Vγ9 (clone B3, BD), CD27 (clone M‐T271, BD), CD45RA (clone HI100, BD), PD‐1 (clone EH12.1, BD), CCR7 (clone, 3D12, BD), FasL (clone NOK‐1, BD), CXCR3 (clone 1C6/CXCR3, BD), perforin (clone δG9, BD), granzyme B (clone GB11, BD), NKp46 (clone 9E2/NKp46, BD), NKG2D (clone 1D11, BD), HLA‐DR (clone L243, Biolegend), CD25 (clone M‐A251), CD107a (clone H4A3, Biolegend), IFN‐γ (clone 4S.B3, BD), TNF‐α (clone Mab11, BD), IL‐4 (clone 8D4‐8, BD), IL‐17A (clone N49‐653, BD), 4‐1BB (clone 4B4‐1, BD), OX40 (clone ACT35, BD), BTN3A1 (clone eBioBT3.1, Invitrogen) and anti‐FC (clone EM‐07, Invitrogen). The mouse CD45 (clone 30‐F11, BD) monoclonal antibody used for flow cytometry was purchased from BD bioscience. The zombie dye (423 102) was purchased from Biolegend. The apoptosis kit (559 925) was purchased from BD biosciences.

To test the infiltrating γδ T cells in tumor tissue or the circulating γδ T cells in peripheral blood, flow cytometric analysis was performed using LSR Fortessa cytometer (BD). Tumor tissue was dissociated and digested (gentle MACS Dissociator, Miltenyi Biotec) to produce single‐cell suspension for flow detection. To test the proportion and surface characteristics of γδ T cells, single cells of tumor tissue or PBMC were incubated with a zombie dye (423 102, Biolegend) to identify dead/live cells and with Fc receptor blocking solution (Cat 422 302, Biolegend) for 15 min, and then the cells were incubated for 30 min at 4 °C with a mixture of fluorescein‐labeled antibodies. For intracellular staining, single cells fixation and permeabilization were performed after surface protein staining, and then the intracellular proteins were stained by a mixture of fluorescein‐labeled antibodies. When analyzing the flow cytometry data, dead cells were excluded based on zombie dye staining, and all live cells were then gated based on forward (FSC‐A, measuring cell size) and side (SSC‐A, measuring cell granularity) scatter to exclude debris, followed by gating for single cells by FSC‐A versus FSC‐W parameters. Tumor‐infiltrating γδ T cells were identified as CD45^+^CD3^+^TCR γδ^+^, CD45^+^CD3^+^TCR γδ^+^Vδ1^+^, CD45^+^CD3^+^TCR γδ^+^Vδ2^+^. Peripheral circulating and in vitro expanded γδ T cells were identified as CD45^+^CD3^+^TCR γδ^+^, CD45^+^CD3^+^TCR γδ^+^Vδ1^+^ and CD45^+^CD3^+^TCR γδ^+^Vγ9^+^Vδ2^+^. Vγ9Vδ2 T cell activation, differentiation and killing potential were determined by surface protein expression of CCR7, HLA‐DR, CXCR3, NKG2D, NKp46, PD‐1, CD16, CD40, CD54, CD56, CD86, CD107a, FasL and intracellular expression of IFN‐γ, TNF‐α, perforin and granzyme B.

For detection of the antigen uptake and processing capacity of expanded Vγ9Vδ2 T cells, cells were cocultured with DQ‐OVA (D12053, Invitrogen) in the presence or absence of chloroquine (CQ, C6628, Sigma‐aldrich) for 6 h. The proportion of DQ fluorescence positive cells and the intensity of DQ fluorescence were determined by flow cytometry. The LysoTracker (Cat L7528) was purchased from Invitrogen.

For detection of Vγ9Vδ2 T cell induced Mart‐1_27‐35_ specific TCR‐T cell activation, control Vγ9Vδ2 T cells or Mart‐1 long peptide (MPREDAHFIYGYPKKGHGHSYTTAEEAAGIGILTVILGVLLLIGCWYCRRRNGYRALMDKSLHVGTQCALTRRCPQEGFDHRDSKVSLQEKNCEPVVPNAPPAYEKLSAEQSPPPYSP)‐loaded or Mart‐1 vector transfected Vγ9Vδ2 T cells were cocultured with Mart‐1_27‐35_ specific TCR‐T cells or control Pan‐T cells for 24 h, then the cells were collected and stained with zombie dye, Fc receptor blocking solution, surface protein 4‐1BB, OX40, and determined by flow cytometry.

For detection of Vγ9Vδ2 T cell induced naïve αβ T cell (HLA‐matched or partially matched) activation, control Vγ9Vδ2 T cells or CCLs‐loaded Vγ9Vδ2 T cells were cocultured with naïve αβ T cells for 72 h or 120 h. In the 72‐hour cocultures, cells were collected and stained with zombie dye, Fc receptor blocking solution, surface protein 4‐1BB, OX40, and determined by flow cytometry. In the 120‐hour cocultures, cells were collected and stained with zombie dye, Fc receptor blocking solution, intracellular protein IFN‐γ, TNF‐α, Granzyme B and determined by flow cytometry.

### In Vitro BTN3A1‐Fc Binding Assays

To determine the influence of Vγ9Vδ2 T cell on the binding between BTN3A1 and αβ T cells, 1 × 10^6^ primary αβ T cells were incubated with BTN3A1‐Fc (10 µg mL^−1^, 8539‐BT, R&D) in the presence or absence of irradiated Vγ9Vδ2 T cells (1 × 10^6^, Fc receptor blocking solution pretreated) for 30 min at 4 °C. To verify the effect of Vγ9Vδ2 T cells, a separate group was added with TCR‐Vγ9 blocking antibody. After incubation, cell pellets were collected and subsequently subjected to zombie dye, Fc receptor blocking solution, anti‐CD3/CD4/CD8/TCR γδ and anti‐Fc antibodies staining and flow cytometry determination.

To determine the potential of Vγ9Vδ2 T cells to inhibit BTN3A1‐Fc binding to primary αβ T cells, irradiated Vγ9Vδ2 T cells (Fc receptor blocking solution pretreated) and primary αβ T cells were added to 96‐well plates in different ratios (1:0, 10:1, 5:1 and 1:1) in the presence of plate‐bound OKT3 (10 µg mL^−1^, Cat 317 326, Biolegend), and BTN3A1‐Fc (10 µg mL^−1^, R&D) or control Fc protein was added simultaneously. After incubating at 4 °C for 30 min, cell pellets were collected and subsequently subjected to zombie dye, Fc receptor blocking solution, anti‐CD3/CD4/CD8 and anti‐Fc antibodies staining and flow cytometry determination.

### In Vitro αβ T Cell Proliferation Assays

To explore whether Vγ9Vδ2 T cell can reverse BTN3A1‐mediated suppression of αβ T cell response: 1) 1 × 10^6^ primary αβ T cells (CTV labeled, C34557, Invitrogen) were activated in the presence of plate‐bound CD3 (OKT3, 10 µg mL^−1^, Cat 317 326, Biolegend), CD28 (CD28.2, 250 ng mL^−1^, Cat 317 326, Biolegend) antibodies and BTN3A1‐Fc (10 µg mL^−1^, R&D); 2) 1 × 10^6^ primary αβ T cells (CTV labeled) were activated in the presence of plate‐bound CD3 (OKT3, 10 µg mL^−1^, Cat 317 326, Biolegend), CD28 (CD28.2, 250 ng mL^−1^, Cat 317 326, Biolegend) antibodies and mitomycin C (20 µg mL^−1^, HY‐13316, MCE) pretreated K562‐BTN3A1^OE^ cells (1 × 10^6^); To verify the effect of Vγ9Vδ2 T cells, 1 × 10^6^ irradiated (50 Gy) Vγ9Vδ2 T cells (anti‐CD16/32 pretreated) with or without TCR γδ blocking antibody were added simultaneously. The anti‐TCR γδ blocking antibody (clone B1, 331 202) or (clone 7A5, TCR1720) was purchased from Biolegend and Invitrogen, respectively. After 96 hours, cell pellets and supernatants were collected. Cells were subjected to zombie dye, Fc receptor blocking solution, anti‐CD3/CD4/CD8 antibodies staining and flow cytometry determination. The concentration of IFN‐γ in the supernatants was determined by ELISA kit (430 104, Biolegend).

### In Vitro Measurement of TCR‐T Cell‐Mediated CaSki Cell Death

To explore whether Vγ9Vδ2 T cell can reverse BTN3A1‐mediated suppression of αβ T cell killing capacity, 1 × 10^6^ Mart‐1_27‐35_ specific TCR‐T cells were cocultured with Mart‐1_27‐35_ peptides loaded CaSki‐vector or CaSki‐BTN3A1^OE^ cells (2 × 10^6^, HLA‐A2^+^) for 12 h. To verify the effect of Vγ9Vδ2 T cells, 1 × 10^6^ irradiated Vγ9Vδ2 T cells with or without TCR γδ blocking antibody were added simultaneously. After incubation, the cells were collected and subsequently subjected to cell death determination by flow cytometry.

### Surface BTN3A1 Expression Assays

To determine the surface BTN3A1 expression level on CC cell lines, SiHa, HeLa, C‐33A, CaSki and control cell line HUVEC, or the transfected cell lines including K562‐vector, K562‐BTN3A1^OE^, CaSki‐vector, CaSki‐BTN3A1^OE^ were stained with anti‐BTN3A1 mAb (Invitrogen, clone eBioBT3.1, 14) or isotype control (Invitrogen, 14) at 4 °C for 30 min, and then cells were subjected to flow cytometry determination.

To determine the surface BTN3A1 expression level on CC cells after IFN‐γ stimulation, CC cell lines (CaSki, Siha and HeLa) were treated with different concentrations of IFN‐γ (0, 10, 100, and 1000 ng mL^−1^, 570 202, Biolegend) for 24 h, cells pellets were collected and subsequently subjected to anti‐BTN3A1 mAb (Invitrogen, clone eBioBT3.1, 14) or isotype control (Invitrogen, 14) staining and flow cytometry determination.

To determine the mechanism of IFN‐γ‐induced BTN3A1 expression on CC cells, STAT1 inhibitor (Fludarabine, HY‐B0069, MCE), NF‐κB inhibitor (JSH‐23, HY‐13982, MCE) and ERK1/2 inhibitor (SCH772984, HY‐112570, MCE) were added to the IFN‐γ (100 ng mL^−1^) treated culture system. After 24 h, cells pellets were collected and subsequently subjected to anti‐BTN3A1 mAb (Invitrogen, clone eBioBT3.1, 14) or isotype control (Invitrogen, 14) staining and flow cytometry determination.

To determine the surface BTN3A1 expression level on CC cell induced by activated αβ T cell‐derived IFN‐γ, Mart‐1_27‐35_ peptides‐loaded CaSki cell (2 × 10^6^, HLA‐A2^+^) were cocultured with Mart‐1_27‐35_ specific TCR‐T cells (1 × 10^6^) in the absence or presence of IFN‐γ blocking antibody for 48 hours. The intensity of surface BTN3A1 on CaSki cells was determined by flow cytometry. The IFN‐γ blocking antibody (clone B27, 506 531) was purchased from Biolegend.

### Bioinformatics Analysis

CESC RNA‐seq data was downloaded based on Illumina platform from the Cancer Genome Atlas (TCGA) database (https://portal.gdc.cancer.gov/), which including 306 cervical squamous cell carcinoma and endocervical adenocarcinoma samples. Two types of the RNA‐seq data, including raw counts and fragments per kilo base per million mapped (FPKM) reads, were applied to the survival, immune infiltration correlation, cytolytic activity score, and gene expression correlation analyses. RNA‐seq data were converted from FPKM (fragments per kilobase per million) format to TPM (transcripts per million reads) format and log2‐transformed.

Among the bioinformatics analyses, immune infiltration correlation, cytolytic activity score, and gene expression correlation analysis in CC was mapped using the TIMER2.0 database,^[^ ^89^ ^]^ Kaplan‐Meier plotter database^[^ ^49^ ^]^ and XIANTAO tools platform (https://www.xiantao.love/, accessed on December 3, 2022). To examine the correlation of *TRGV9* and *TRDV1* with CC patient prognosis, gene expression data analyzed by XIANTAO tools platform and the prognostic data from a Cell press article^[^ ^90^ ^]^ were used. The correlation of γδ T cell infiltration with CC patient prognosis was analyzed by TIMER2.0 database (http://timer.cistrome.org/, accessed on August 1, 2022). Cytolytic activity score (CYT) score was calculated using the log‐transformed geometric mean of *GZMA* and *PRF1* TPM values.

### RNA Sequencing of Peripheral Vγ9Vδ2 T Cells from CC Patients and HC Subjects

Vγ9Vδ2 T cells were purified from the PBMC of healthy donors and CC patients with or without neoadjuvant chemotherapy. Total RNA was isolated and mRNA libraries were prepared using the Illumina technology. RNA sequencing and sequence quality control were performed by BGI‐SEQ platform. The detailed analysis, including gene heatmap, gene set enrichment analysis (GSEA), and gene annotation of the sequencing data was completed by BGI Dr. Tom system.

### Immunofluorescence Analysis and Imaging

For immunofluorescence experiments, OVA‐DQ pretreated Vγ9Vδ2 T cells (1 × 10^6^) were fixed with 4% paraformaldehyde and permeabilized with 0.1% Triton‐X/PBS. After washing, cells were sequentially stained with 100 nM LysoTracker (Invitrogen) and DAPI, according to the manufacturer's instructions. The cells were mounted in VectaShield (Vector Laboratories, Burlingame, CA, USA) and examined using a confocal scanning laser microscope.

### Generation of Tumor‐Infiltrating Lymphocytes (αβ TILs)

TILs used for in vitro experiments and adoptive transfer were generated as previously described.^[^ ^88^ ^]^ Briefly, surgically resected CC (patient CAT039) tissues were collected and cut into approximately 1 mm fragments in size and placed individually into 24‐well plate containing 2 mL of complete media in the presence of rhIL‐2 (6000 IU mL^−1^, GeneScript). Complete media were consisted of RPMI 1640 with 10% human AB serum, 2 mM L‐glutamine, 25 mM HEPES and 10 µg mL^−1^ gentamicin supplementation. For adoptive transfer, some TIL cultures were selected for rapidly expansion after the initial outgrowth in the presence of irradiated PBMC at a ratio of 1 to 100. The medium was consisted of a 1 to 1 mixture of complete RPMI and AIM‐V with 5% human AB serum, 3000 IU mL^−1^ IL‐2, and 30 ng mL^−1^ OKT3 antibody (Biolegend) supplementation. All cells were cultured at 37 °C with 5% CO_2_ condition.

### Construction of Mart‐1_27‐35_ Specific DMF5 TCR‐T Cells

As described previously,^[^ ^91^ ^]^ cDNA sequences encoding DMF5 TCR were synthesized and cloned into a pLV lentiviral vector. A single porcine teschovirus derived 2A sequence (P2A) was used to link the TCR genes in a β‐P2A‐α configuration. These TCR transgene cassettes were codon optimized to maximize DMF5 TCR expression and pairing. Lentivirus particle was produced by 293T cells transfected with the pMD2.G, psPAX2 and pLV vectors. To generate lentivirus supernatants, the pLV vector encoding the DMF5 TCR (2 µg per well) and the assisting plasmids pMD2.G and psPAX2 (1 µg per well) were co‐transfected into the packaging cell line 293T (1 × 10^6^ cells per well in 6‐well plates coated with poly‐D‐lysine) in the presence of Lipofectamine 3000 (Thermo). Lentiviral supernatants were collected at 48 h after transfection. Primary human αβ T cells were then engineered to express the DMF5 TCR sequence through lentivirus transfection and therefore could recognize the Mart‐1_27‐35_ peptide antigen presented by the Vγ9Vδ2 T cells or cervical cancer CaSki cells. Briefly, healthy donor‐derived PBMCs were stimulated and activated with anti‐CD3 (5 µg mL^−1^, clone OKT3, Cat 317 326, BioLegend) and CD28 (5 µg mL^−1^, clone CD28.2, Cat 302 934, BioLegend) antibodies in the presence of 200 IU mL^−1^ human recombinant IL‐2. After 48 h, activated αβ T cells were transduced with DMF5 TCR virus to generate DMF5 TCR‐T cells. DMF5 TCR‐T cells were cultured in complete human T cell medium (RPMI 1640 supplemented with 10% FBS, non‐essential amino acids, L‐glutamine, HEPES and sodium pyruvate) in the presence of 200 IU mL^−1^ rhIL‐2.

### Neoantigens Prediction and In Vitro Validation

Tumor tissues and peripheral blood of patient CAT039 were collected and used to extract DNA and/or RNA. Tissue‐ and peripheral blood‐derived DNA were subjected to whole exon sequencing (WES) and RNA was used for transcriptomic sequencing (RNA‐seq). WES and RNA‐seq were conducted by the Yuce Biotechnology Co., Ltd.

As described previously,^[^ ^88^ ^]^ tumor cell neoantigen was predicted through the somatic mutation identification, HLA typing, algorithm prediction of affinity between mutation peptides and HLA (HLA‐I and HLA‐II) of patients and neoantigen score filtering. Peptide‐HLA binding affinity was determined through the Immune Epitope Database and Analysis Resource (IEDB)‐T cell prediction tools (version 2.5) and TruNeo algorithm. All variant‐containing 8‐11‐mers peptides for major histocompatibility complex (MHC) class I or 12‐15‐mers peptides for MHC class II were included. The affinity between peptide and HLAs <500 nM was defined as candidate neoepitopes for subsequent filtering. The candidate neoepitopes were filtered based on a predefined set of criteria: (1) strong binding peptides were shown as IC50 < 50 nM or %Rank < 0.5; (2) mutated peptides showed a higher affinity than the matched wildtype peptides; (3) mutations showed a higher tumor variant allele fraction; (4) mutated peptides showed a high expression status, with at least > 5 reads cover mutated allele in RNA‐seq data; and (5) oncogene mutations were included. Based on these criteria, a total of 12 predicted peptides for patient CAT039 were selected for peptide synthesis (each peptide is 29 aa in length with the aa residue generated by nonsynonymous mutation put in the middle, which included both MHC I and II epitopes) and in vitro validation. The predicted neoantigens were co‐incubated with autologous EBV‐LCL cells or expanded Vγ9Vδ2 T cells in vitro, and then these two types of cells were used to induce autologous αβ TIL activation, respectively. After 24 h, IFN‐γ ELISPOT (CT230, U‐Cytech) assay and flow cytometry for detection of αβ TIL surface activation markers (4‐1BB and OX40) were conducted.

### Preclinical Models

Female NOD/SCID/IL2Rγ^−/−^ (NCG; NOD/ShiLtJGpt‐Prkdc^em26Cd52^ Il2rg^em26Cd22^ /Gpt) mice, aged 6 weeks, were purchased from Gempharmatech Co., Ltd (Nanjing, China). These experimental mice were housed in isolator cages maintained under specific pathogen free conditions, with precisely regulated temperature and humidity, and a standardized 12‐hour light/dark cycle. All animal handling, surveillance, and experimentation were strictly adhered to the guidelines for ethical review of animal welfare and were approved by the Institutional Animal Welfare and Ethic Committee at Huazhong University of Science and Technology.

For subcutaneous SiHa and HeLa CC models, 6 weeks old female NCG mice were used. 2 × 10^6^ SiHa or HeLa cells were inoculated subcutaneously in NCG mice. When tumors were grown to palpable, mice bearing tumors were randomized into different groups to ensure a similar mean tumor burden per group, and freshly expanded Vγ9Vδ2 T cells (1 × 10^7^ cells per mouse) or control medium were injected into the tumor‐bearing mice through tail vein. Five days later, the CCLs unloaded or loaded Vγ9Vδ2 T cell stimulated αβ T cell culture products (the ratio of CD4^+^ to CD8^+^ T cell was ≈1.5:1, showed in Figure S5a,b, Supporting Information) were mixed with fresh autologous CCLs unloaded or loaded Vγ9Vδ2 T cells at a 1:1 ratio, and then the cell mixtures were injected into the tumor‐bearing mice through tail vein (1 × 10^7^ cell mixtures per mouse). Tumor growth was monitored continuously, and the survival of the mice was recorded. Mice with tumors larger than 2000 mm^3^ were considered to have reached the end point and were euthanized and marked as dead in the survival record. For the γδ/αβ T cell and chemotherapy combination experiments, SiHa cell tumor models in NCG mice were established and treated with cis‐platin (cDDP, *i.p*., 5 mg kg^−1^ body weight) twice after being palpable, followed by γδ T cell (1 × 10^7^ cells per mouse) and the Vγ9Vδ2 T cell and in vitro stimulated αβ T cell mixture products (in the absence or presence of CCLs; 1 × 10^7^ cells per mouse) or control medium treatments.

In some cases, the experiment was terminated when there was a large difference in tumor growth among the groups of mice. After the mice were euthanized, tumor tissues and major organs were collected, and peripheral blood was obtained. Tumor tissue was used for preparation of single‐cell suspension and immunofluorescence assay. Single‐cell suspension was used to detect the infiltration (CD45^+^CD3^+^CD4^+^/CD8/γδ TCR^+^) and cytokine (IFN‐γ, TNF‐α) secretion of the transferred T cells in tumor tissues by flow cytometry. In immunofluorescence assay, T cell marker (CD4^+^, CD8^+^) and activation molecules (HLA‐DR, CD107a) staining was used to analyze the infiltration and activation levels of transferred T cells in tumor tissues. The human antibodies used for immunofluorescence assays are as follows: HLA‐DR (clone G‐7, Santa cruz biotechnology; clone EPR3692, abcam), CD4 (clone EPR6855, abcam), CD8 (clone CAL66, abcam), TCR Vδ2 (clone B6, Biolegend), CD107a (clone H4A3, BD). Peripheral blood was prepared for PBMC, and the distribution of transferred T cells (CD45^+^CD3^+^CD4^+^/CD8/γδ TCR^+^) in PBMC was analyzed by flow cytometry. The main organs of mice were used for HE staining to explore the changes and injuries of the organs.

For patient‐derived xenograft (PDX) models, 6 weeks old female NCG mice were used again. Tumor tissue from CC patient CAT039 was cut to 3 mm in size after antibiotics treatment, and then tumor tissues were inoculated subcutaneously in NCG mice. Mice bearing engrafted PDX tumors were randomly assigned into groups, to ensure a similar mean tumor volume per group (≈60 mm^3^). Each group of mice was given freshly expanded αβ TIL or Vγ9Vδ2 T cells (1 × 10^7^ cells per mouse, Vγ9Vδ2 T cells were loaded with in vitro validated neoantigen peptide pools or wild type peptide pools) or control medium; After 2 and 8 days, αβ TIL or Vγ9Vδ2 T cells (loaded with neoantigen peptide pools or wild type peptide pools) and αβ TIL mixed cell products (1:1, 5 × 10^6^ γδ T versus 5 × 10^6^ αβ TIL) were transferred twice. Tumor growth was monitored continuously, and the survival of the mice was recorded. In addition, PDX tumor tissues were collected for immunofluorescence detection of the infiltration (TCR Vδ2^+^, CD4^+^, CD8^+^) and activation (HLA‐DR, CD107a) levels of transferred T cells.

### IHC Staining of BTN3A1

Tumor specimens and adjacent tissues from CC patients were fixed in 4% formalin, embedded in paraffin, and stained with ant‐BTN3A1 Ab according to manufacturer recommendations. Briefly, tissue sections were incubated in Tris EDTA buffer at 95 °C for 1 hour to retrieve antigenicity, followed by incubation with anti‐BTN3A1 Ab (PA5‐97513, Invitrogen) at 1:100 for 1 h. Slides were then incubated with the respective secondary antibody with 1:500 dilutions, followed by HRP and DAB detection.

### Quantification and Statistical Analysis

Data analysis and visualization were conducted using Prism 9 (Graphpad software). Graphs represent mean values ± SEM or SD as indicated in the figure legends. Statistical tests were two‐tailed Student's *t* test or one‐way multiple comparisons ANOVA with Tukey's multiple comparison test when more than 2 groups were compared. Kaplan‐Meier survival curves of in vivo tumor experiments were analyzed using log‐rank test. *p* < 0.05 was considered statistically significant and *p* values are denoted with asterisks: ****p < 0.0001, ***p < 0.001, **p < 0.01, *p < 0.05) and NS means *p* > 0.05.

## Conflict of Interest

The authors declare no conflict of interest.

## Author Contributions

M.W., J.L., and L.L. contributed equally to this work. M.W., Y.H., H.W., and W.Z. conceived the idea, designed the experiments, and composed the paper. M.W. conducted all the experiments. J.L., L.L., Y.Y., H.L., H.L., H.J., and S.Q. assisted in animal experiments. L.Y., and H.Z. assisted in flow cytometry analysis. J.L. and L.L. assisted in cell culture and in vitro experiments; J.L., L.L., Y.C., and J.W. assisted in tissue cultures. Y.Y., S.Y., R.X., L.W., and Y.Z. assisted in bioinformatics analysis. M.W., Y.H., H.W., W.Z., and J.H. contributed to the interpretation of the results. Y.H., H.W., and W.Z. supervised the project.

## Supporting information



Supporting Information

## Data Availability

The raw sequence data reported in this paper have been deposited in the Genome Sequence Archive (Genomics, Proteomics & Bioinformatics 2021)^[^ ^92^ ^]^ in National Genomics Data Center (Nucleic Acids Res 2022),^[^ ^93^ ^]^ China National Center for Bioinformation/Beijing Institute of Genomics, Chinese Academy of Sciences (GSA‐Human: HRA006062 for Vγ9Vδ2 T cell transcriptomes data showed in Figures 2, 3, and 6; HRA005334 for RNA sequencing data of cervical cancer tumor tissue showed in Figure 7b) that are publicly accessible at https://ngdc.cncb.ac.cn/gsa‐human. Human cervical cancer cohort RNA expression data were derived from the TCGA Research Network: https://portal.gdc.cancer.gov. All other data supporting the findings of this study are available from the corresponding author upon reasonable request. Source data are provided with this paper. No custom algorithms were used in this study. The R code that was used to perform differential expression analysis on the TCGA cohort can be found on XIANTAO tools platform (https://www.xiantao.love/).

## References

[1] H. Sung , J. Ferlay , R. L. Siegel , M. Laversanne , I. Soerjomataram , A. Jemal , F. Bray , Ca‐Cancer J. Clin. 2021, 71, 209. 33538338 10.3322/caac.21660

[2] K. S. Pfaendler , K. S. Tewari , Am. J. Obstet. Gynecol. 2016, 214, 22. 26212178 10.1016/j.ajog.2015.07.022 PMC5613936

[3] S. Bagchi , R. Yuan , E. G. Engleman , Annu. Rev. Pathol. 2021, 16, 223. 33197221 10.1146/annurev-pathol-042020-042741

[4] A. V. Finck , T. Blanchard , C. P. Roselle , G. Golinelli , C. H. June , Nat. Med. 2022, 28, 678. 35440724 10.1038/s41591-022-01765-8 PMC9305718

[5] B. J. Monk , T. Enomoto , W. M Kast , M. McCormack , D. S. P. Tan , X. Wu , A. González‐Martín , Cancer Treat. Rev. 2022, 106, 102385. 35413489 10.1016/j.ctrv.2022.102385 PMC10697630

[6] H. C. Chung , W. Ros , J.‐P. Delord , R. Perets , A. Italiano , R. Shapira‐Frommer , L. Manzuk , S. A. Piha‐Paul , L. Xu , S. Zeigenfuss , S. K. Pruitt , A. Leary , J. Clin. Oncol. 2019, 37, 1470. 30943124 10.1200/JCO.18.01265

[7] J.‐S. Frenel , C. Le Tourneau , B. O'Neil , P. A. Ott , S. A. Piha‐Paul , C. Gomez‐Roca , E. M. J. van Brummelen , H. S. Rugo , S. Thomas , S. Saraf , R. Rangwala , A. Varga , J. Clin. Oncol. 2017, 35, 4035. 29095678 10.1200/JCO.2017.74.5471

[8] A. S. Matos , M. Invenção , I. A. Moura , A. C. Freitas , M. V. A. Batista , Rev. Med. Virol. 2023, 33, e2463. 37291746 10.1002/rmv.2463

[9] J. W. Youn , S.‐Y. Hur , J. W. Woo , Y.‐M. Kim , M. C. Lim , S. Y. Park , S. S. Seo , J. H. No , B.‐G. Kim , J.‐K. Lee , S. J. Shin , K. Kim , M. F. Chaney , Y.‐J. Choi , Y. S. Suh , J. S. Park , Y. C. Sung , Lancet Oncol. 2020, 21, 1653.33271094

[10] L. Ferrall , K. Y. Lin , R. B. S. Roden , C. F. Hung , T. C. Wu , Clin. Cancer Res. 2021, 27, 4953 . 33888488 10.1158/1078-0432.CCR-20-2833 PMC8448896

[11] S. L. Doran , S. Stevanovic , S. Adhikary , J. J. Gartner , L. Jia , M. L. M. Kwong , W. C. Faquin , S. M. Hewitt , R. M. Sherry , J. C. Yang , S. A. Rosenberg , C. S. Hinrichs , J. Clin. Oncol. 2019, 37, 2759. 31408414 10.1200/JCO.18.02424 PMC6800280

[12] N. B. Nagarsheth , S. M. Norberg , A. L. Sinkoe , S. Adhikary , T. J. Meyer , J. B. Lack , A. C. Warner , C. Schweitzer , S. L. Doran , S. Korrapati , S. Stevanovic , C. L. Trimble , J. A. Kanakry , M. H. Bagheri , E. Ferraro , S. H. Astrow , A. Bot , W. C. Faquin , D. Stroncek , N. Gkitsas , S. Highfill , C. S. Hinrichs , Nat. Med. 2021, 27, 419. 33558725 10.1038/s41591-020-01225-1 PMC9620481

[13] A. A. Jazaeri , E. Zsiros , R. N. Amaria , A. S. Artz , R. P. Edwards , R. M. Wenham , B. M. Slomovitz , A. Walther , S. S. Thomas , J. A. Chesney , R. Morris , K. Matsuo , S. Gaillard , P. G. Rose , J. G. Donas , J. M. Tromp , F. Tavakkoli , H. Li , M. Fardis , B. J. Monk , J. Clin. Oncol. 2019, 37, 2538.

[14] S. Stevanovic , L. M. Draper , M. M. Langhan , T. E. Campbell , M. L. Kwong , J. R. Wunderlich , M. E. Dudley , J. C. Yang , R. M. Sherry , U. S. Kammula , N. P. Restifo , S. A. Rosenberg , C. S. Hinrichs , J. Clin. Oncol. 2015, 33, 1543. 25823737 10.1200/JCO.2014.58.9093 PMC4417725

[15] S. Stevanovic , S. R. Helman , J. R. Wunderlich , M. M. Langhan , S. L. Doran , M. L. M. Kwong , R. P. T. Somerville , C. A. Klebanoff , U. S. Kammula , R. M. Sherry , J. C. Yang , S. A. Rosenberg , C. S. Hinrichs , Clin. Cancer Res. 2019, 25, 1486. 30518633 10.1158/1078-0432.CCR-18-2722 PMC6397671

[16] G. Oliveira , C. J. Wu , Nat. Rev. Cancer 2023, 23, 295. 37046001 10.1038/s41568-023-00560-y PMC10773171

[17] A. D. Waldman , J. M. Fritz , M. J. Lenardo , Nat. Rev. Immunol. 2020, 20, 651. 32433532 10.1038/s41577-020-0306-5 PMC7238960

[18] K. F. Bol , G. Schreibelt , K. Rabold , S. K. Wculek , J. K. Schwarze , A. Dzionek , A. Teijeira , L. E. Kandalaft , P. Romero , G. Coukos , B. Neyns , D. Sancho , I. Melero , I. J. M. de Vries , J. Immunother. Cancer 2019, 7, 109. 30999964 10.1186/s40425-019-0580-6 PMC6471787

[19] E. W. Roberts , M. L. Broz , M. Binnewies , M. B. Headley , A. E. Nelson , D. M. Wolf , T. Kaisho , D. Bogunovic , N. Bhardwaj , M. F. Krummel , Cancer Cell 2016, 30, 324. 27424807 10.1016/j.ccell.2016.06.003 PMC5374862

[20] S. K. Wculek , F. J. Cueto , A. M. Mujal , I. Melero , M. F. Krummel , D. Sancho , Nat. Rev. Immunol. 2020, 20, 7. 31467405 10.1038/s41577-019-0210-z

[21] A. A. Shamseddine , B. Burman , N. Y. Lee , D. Zamarin , N. Riaz , Cancer Discov. 2021, 11, 1896. 33990345 10.1158/2159-8290.CD-20-1760 PMC8338882

[22] D. Kabelitz , R. Serrano , L. Kouakanou , C. Peters , S. Kalyan , Cell. Mol. Immunol. 2020, 17, 925. 32699351 10.1038/s41423-020-0504-x PMC7609273

[23] S. Mensurado , R. Blanco‐Domínguez , B. Silva‐Santos , Nat. Rev. Clin. Oncol. 2023, 20, 178. 36624304 10.1038/s41571-022-00722-1

[24] B. Silva‐Santos , S. Mensurado , S. B. Coffelt , Nat. Rev. Cancer 2019, 19, 392. 31209264 10.1038/s41568-019-0153-5 PMC7614706

[25] A. J. Gentles , A. M. Newman , C. L. Liu , S. V. Bratman , W. Feng , D. Kim , V. S. Nair , Y. Xu , A. Khuong , C. D. Hoang , M. Diehn , R. B. West , S. K. Plevritis , A. A. Alizadeh , Nat. Med. 2015, 21, 938. 26193342 10.1038/nm.3909 PMC4852857

[26] Z. Sebestyen , I. Prinz , J. Déchanet‐Merville , B. Silva‐Santos , J. Kuball , Nat. Rev. Drug Discovery 2020, 19, 169. 31492944 10.1038/s41573-019-0038-z

[27] M. Brandes , K. Willimann , B. Moser , Science 2005, 309, 264. 15933162 10.1126/science.1110267

[28] M. Brandes , K. Willimann , G. Bioley , N. Lévy , M. Eberl , M. Luo , R. Tampé , F. Lévy , P. Romero , B. Moser , Proc. Natl. Acad. Sci. USA 2009, 106, 2307. 19171897 10.1073/pnas.0810059106 PMC2650152

[29] S. Meuter , M. Eberl , B. Moser , Proc. Natl. Acad. Sci. USA 2010, 107, 8730. 20413723 10.1073/pnas.1002769107 PMC2889313

[30] M. W. Khan , M. Eberl , B. Moser , Front. Immunol. 2014, 5, 512. 25374569 10.3389/fimmu.2014.00512 PMC4204533

[31] K. Yoshihara , M. Shahmoradgoli , E. Martínez , R. Vegesna , H. Kim , W. Torres‐Garcia , V. Treviño , H. Shen , P. W. Laird , D. A. Levine , S. L. Carter , G. Getz , K. Stemke‐Hale , G. B. Mills , R. G. W. Verhaak , Nat. Commun. 2013, 4, 2612. 24113773 10.1038/ncomms3612 PMC3826632

[32] S. Hänzelmann , R. Castelo , J. Guinney , BMC Bioinformatics 2013, 14, 7. 23323831 10.1186/1471-2105-14-7 PMC3618321

[33] M. S. Rooney , S. A. Shukla , C. J. Wu , G. Getz , N. Hacohen , Cell 2015, 160, 48. 25594174 10.1016/j.cell.2014.12.033 PMC4856474

[34] C. S. Eberhardt , H. T. Kissick , M. R. Patel , M. A. Cardenas , N. Prokhnevska , R. C. Obeng , T. H. Nasti , C. C. Griffith , S. J. Im , X. Wang , D. M. Shin , M. Carrington , Z. G. Chen , J. Sidney , A. Sette , N. F. Saba , A. Wieland , R. Ahmed , Nature 2021, 597, 279. 34471285 10.1038/s41586-021-03862-z PMC10201342

[35] S. Wen , H. Lu , D. Wang , J. Guo , W. Dai , Z. Wang , J. Leukoc. Biol. 2021, 110, 585. 34047386 10.1002/JLB.5MR1120-778R

[36] H. W. Lee , Y. S. Chung , T. J. Kim , Immune. Netw. 2020, 20, e5. 32158593 10.4110/in.2020.20.e5 PMC7049581

[37] A. J. Nicol , H. Tokuyama , S. R. Mattarollo , T. Hagi , K. Suzuki , K. Yokokawa , M. Nieda , Br. J. Cancer 2011, 105, 778. 21847128 10.1038/bjc.2011.293 PMC3171009

[38] D. Lee , C. J. Rosenthal , N. E. Penn , Z. S. Dunn , Y. Zhou , L. Yang , Cancers (Basel) 2022, 14, 3005. 35740670 10.3390/cancers14123005 PMC9221220

[39] Y. Xu , Z. Xiang , M. Alnaggar , L. Kouakanou , J. Li , J. He , J. Yang , Yi Hu , Y. Chen , Li Lin , J. Hao , J. Li , J. Chen , M. Li , Q. Wu , C. Peters , Q. Zhou , J. Li , Y. Liang , X. Wang , B. Han , M. Ma , D. Kabelitz , K. Xu , W. Tu , Y. Wu , Z. Yin , Cell. Mol. Immunol. 2021, 18, 427. 32939032 10.1038/s41423-020-0515-7 PMC8027668

[40] L. Li , S. P. Goedegebuure , W. E. Gillanders , Ann. Oncol. 2017, 28, xii11. 29253113 10.1093/annonc/mdx681 PMC5834106

[41] N. Xie , G. Shen , W. Gao , Z. Huang , C. Huang , L. Fu , Signal. Transduct. Target. Ther. 2023, 8, 9. 36604431 10.1038/s41392-022-01270-x PMC9816309

[42] A. Harari , M. Graciotti , M. Bassani‐Sternberg , L. E. Kandalaft , Nat. Rev. Drug Discovery 2020, 19, 635. 32764681 10.1038/s41573-020-0074-8

[43] S. A. Rosenberg , N. P. Restifo , Science 2015, 348, 62. 25838374 10.1126/science.aaa4967 PMC6295668

[44] Y. Tang , Y. Wang , J. Wang , M. Li , L. Peng , G. Wei , Y. Zhang , J. Li , Z. Gao , BMC Bioinformatics 2020, 21, 532. 33208106 10.1186/s12859-020-03869-9 PMC7672179

[45] J. Y. Li , Y. P. Chen , Y. Q. Li , N. Liu , J. Ma , Mol. Cancer 2021, 20, 27. 33541368 10.1186/s12943-021-01317-7 PMC7863268

[46] S. Zhu , T. Zhang , L. Zheng , H. Liu , W. Song , D. Liu , Z. Li , C.‐X. Pan , J. Hematol. Oncol. 2021, 14, 156. 34579759 10.1186/s13045-021-01164-5 PMC8475356

[47] D. R. Principe , S. D. Kamath , M. Korc , H. G. Munshi , Pharmacol. Ther. 2022, 236, 108111. 35016920 10.1016/j.pharmthera.2022.108111 PMC9271143

[48] K. K. Payne , J. A. Mine , S. Biswas , R. A. Chaurio , A. Perales‐Puchalt , C. M. Anadon , T. L. Costich , C. M. Harro , J. Walrath , Q. Ming , E. Tcyganov , A. L. Buras , K. E. Rigolizzo , G. Mandal , J. Lajoie , M. Ophir , J. Tchou , D. Marchion , V. C. Luca , P. Bobrowicz , B. McLaughlin , U. Eskiocak , M. Schmidt , J. R. Cubillos‐Ruiz , P. C. Rodriguez , D. I. Gabrilovich , J. R. Conejo‐Garcia , Science 2020, 369, 942. 32820120 10.1126/science.aay2767 PMC7646930

[49] Á. Nagy , G. Munkácsy , B. Győrffy , Sci. Rep. 2021, 11, 6047. 33723286 10.1038/s41598-021-84787-5 PMC7961001

[50] N. Messal , E. Mamessier , A. Sylvain , J. Celis‐Gutierrez , M.‐L. Thibult , B. Chetaille , G. Firaguay , S. Pastor , Y. Guillaume , Q. Wang , I. Hirsch , J. A. Nunès , D. Olive , Eur. J. Immunol. 2011, 41, 3443. 21918970 10.1002/eji.201141404

[51] T. Yamazaki , I. Goya , D. Graf , S. Craig , N. Martin‐Orozco , C. Dong , J. Immunol. 2010, 185, 5907. 20944003 10.4049/jimmunol.1000835

[52] D. A. Rhodes , W. Reith , J. Trowsdale , Annu. Rev. Immunol. 2016, 34, 151 . 26772212 10.1146/annurev-immunol-041015-055435

[53] Z. Tang , C. Li , B. Kang , G. Gao , C. Li , Z. Zhang , Nucleic Acids Res. 2017, 45, W98. 28407145 10.1093/nar/gkx247 PMC5570223

[54] L. B. Ivashkiv , Nat. Rev. Immunol. 2018, 18, 545. 29921905 10.1038/s41577-018-0029-z PMC6340644

[55] K. Schroder , P. J. Hertzog , T. Ravasi , D. A. Hume , J. Leukoc. Biol. 2004, 75, 163. 14525967 10.1189/jlb.0603252

[56] S. Chen , G. A. Crabill , T. S. Pritchard , T. L. McMiller , P. Wei , D. M. Pardoll , F. Pan , S. L. Topalian , J. Immunother. Cancer 2019, 7, 305. 31730010 10.1186/s40425-019-0770-2 PMC6858680

[57] C. Sun , R. Mezzadra , T. N. Schumacher , Immunity 2018, 48, 434. 29562194 10.1016/j.immuni.2018.03.014 PMC7116507

[58] M. B. Brenner , J. McLean , D. P. Dialynas , J. L. Strominger , J. A. Smith , F. L. Owen , J. G. Seidman , S. Ip , F. Rosen , M. S. Krangel , Nature 1986, 322, 145. 27183647

[59] A. Hayday , Cell 1985, 40, 259. 3917858 10.1016/0092-8674(85)90140-0

[60] A. Bensussan , J. F. Lagabrielle , L. Degos , Blood 1989, 73, 2077. 2525054

[61] S. Ferrini , I. Prigione , S. Mammouti , M. I. Colnaghi , S. Ménard , A. Moretta , L. Moretta , Int. J. Cancer 1989, 44, 245. 2527207 10.1002/ijc.2910440210

[62] E. A. Macintyre , F. Sigaux , Br. J. Haematol. 1989, 73, 2. 2679860 10.1111/j.1365-2141.1989.tb00209.x

[63] R. E. Beatson , A. C. Parente‐Pereira , L. Halim , D. Cozzetto , C. Hull , L. M. Whilding , O. Martinez , C. A. Taylor , J. Obajdin , K. N. Luu Hoang , B. Draper , A. Iqbal , T. Hardiman , T. Zabinski , F. Man , R. T. M. de Rosales , J. Xie , F. Aswad , D. Achkova , C.‐Y. R. Joseph , S. Ciprut , A. Adami , H. G. Roider , H. Hess‐Stumpp , B. Gyorffy , J. Quist , A. Grigoriadis , A. Sommer , A. N. J. Tutt , D. M. Davies , et al., Cell Rep. Med. 2021, 2, 100473. 35028614 10.1016/j.xcrm.2021.100473 PMC8714942

[64] E. Foord , L. C. M. Arruda , A. Gaballa , C. Klynning , M. Uhlin , Sci. Transl. Med. 2021, 13, eabb0192. 33472952 10.1126/scitranslmed.abb0192

[65] N. Zakeri , A. Hall , L. Swadling , L. J. Pallett , N. M. Schmidt , M. O. Diniz , S. Kucykowicz , O. E. Amin , A. Gander , M. Pinzani , B. R. Davidson , A. Quaglia , M. K. Maini , Nat. Commun. 2022, 13, 1372. 35296658 10.1038/s41467-022-29012-1 PMC8927126

[66] S. Chen , Z. Li , W. Huang , Y. Wang , S. Fan , J. Cancer 2021, 12, 4505. 34149914 10.7150/jca.57831 PMC8210570

[67] A. S. Kone , S. Ait Ssi , S. Sahraoui , A. Badou , Int. J. Mol. Sci. 2022, 23, 13424. 36362212 10.3390/ijms232113424 PMC9653866

[68] M. M. Karunakaran , C. R. Willcox , M. Salim , D. Paletta , A. S. Fichtner , A. Noll , L. Starick , A. Nöhren , C. R. Begley , K. A. Berwick , R. A. G. Chaleil , V. Pitard , J. Déchanet‐Merville , P. A. Bates , B. Kimmel , T. J. Knowles , V. Kunzmann , L. Walter , M. Jeeves , F. Mohammed , B. E. Willcox , T. Herrmann , Immunity 2020, 52, 487. 32155411 10.1016/j.immuni.2020.02.014 PMC7083227

[69] L. Yuan , X. Ma , Y. Yang , Y. Qu , X. Li , X. Zhu , W. Ma , J. Duan , J. Xue , H. Yang , J.‐W. Huang , S. Yi , M. Zhang , N. Cai , L. Zhang , Q. Ding , K. Lai , C. Liu , L. Zhang , X. Liu , Y. Yao , S. Zhou , X. Li , P. Shen , Q. Chang , S. R. Malwal , Y. He , W. Li , C. Chen , C.‐C. Chen , et al., Nature 2023, 621, 840. 37674084 10.1038/s41586-023-06525-3 PMC10533412

[70] E. Compte , P. Pontarotti , Y. Collette , M. Lopez , D. F Olive , Eur. J. Immunol. 2004, 34, 2089. 15259006 10.1002/eji.200425227

[71] J. Zhou , J. Zhang , Lu Tao , K. Peng , Q. Zhang , K. Yan , J. Luan , J. Pan , X. Su , J. Sun , Z. Zhang , L. Shen , Proc. Natl. Acad. Sci. USA 2022, 119, e2117523119. 36288286 10.1073/pnas.2117523119 PMC9636952

[72] M. D'Asaro , C. La Mendola , D. Di Liberto , V. Orlando , M. Todaro , M. Spina , G. Guggino , S. Meraviglia , N. Caccamo , A. Messina , A. Salerno , F. Di Raimondo , P. Vigneri , G. Stassi , J. J. Fourniè , F. Dieli , J. Immunol. 2010, 184, 3260. 20154204 10.4049/jimmunol.0903454

[73] S. R. Mattarollo , T. Kenna , M. Nieda , A. J. Nicol , Cancer Immunol. Immunother. 2007, 56, 1285. 17265022 10.1007/s00262-007-0279-2 PMC11030464

[74] N. Joalland , L. Lafrance , T. Oullier , S. Marionneau‐Lambot , D. Loussouarn , U. Jarry , E. Scotet , Oncoimmunology 2019, 8, e1649971. 31646097 10.1080/2162402X.2019.1649971 PMC6791416

[75] J. W. Opzoomer , D. Sosnowska , J. E. Anstee , J. F. Spicer , J. N. Arnold , Front. Immunol. 2019, 10, 1654. 31379850 10.3389/fimmu.2019.01654 PMC6652267

[76] S. A. Patel , A. J. Minn , Immunity 2018, 48, 417. 29562193 10.1016/j.immuni.2018.03.007 PMC6948191

[77] W. D. Yu , G. Sun , J. Li , J. Xu , X. Wang , Cancer Lett 2019, 452, 66. 30902563 10.1016/j.canlet.2019.02.048

[78] S. Srivastava , S. N. Furlan , C. A. Jaeger‐Ruckstuhl , M. Sarvothama , C. Berger , K. S. Smythe , S. M. Garrison , J. M. Specht , S. M. Lee , R. A. Amezquita , V. Voillet , V. Muhunthan , S. Yechan‐Gunja , S. P. S. Pillai , C. Rader , A. M Houghton , R. H. Pierce , R. Gottardo , D. G. Maloney , S. R. Riddell , Cancer Cell 2021, 39, 193. 33357452 10.1016/j.ccell.2020.11.005 PMC7878409

[79] D. C. Deniger , J. S. Moyes , L. J. Cooper , Front. Immunol. 2014, 5, 636. 25566249 10.3389/fimmu.2014.00636 PMC4263175

[80] K. P. Nishimoto , T. Barca , A. Azameera , A. Makkouk , J. M. Romero , Lu Bai , M. M. Brodey , J. Kennedy‐Wilde , H. Shao , S. Papaioannou , A. Doan , C. Masri , N. T. Hoang , H. Tessman , V. D. Ramanathan , A. Giner‐Rubio , F. Delfino , K. Sharma , K. Bray , M. Hoopes , D. Satpayev , R. Sengupta , M. Herrman , S. E. Abbot , B. T. Aftab , Z. An , S. Panuganti , S. M. Hayes , Clin. Transl. Immunology 2022, 11, e1373. 35136603 10.1002/cti2.1373 PMC8809437

[81] W. He , Yi Hu , D. Chen , Y. Li , D. Ye , Q. Zhao , Li Lin , X. Shi , L. Lu , Z. Yin , X. He , Y. Gao , Y. Wu , Clin. Transl. Med. 2022, 12, e800. 35390227 10.1002/ctm2.800 PMC8989380

[82] H. Andrlová , O. Miltiadous , A. I. Kousa , A. Dai , S. DeWolf , S. Violante , H.‐Y. Park , S. Janaki‐Raman , R. Gardner , S. El Daker , J. Slingerland , P. Giardina , A. Clurman , A. L. C. Gomes , C. Nguyen , M. B. da Silva , G. K. Armijo , N. Lee , R. Zappasodi , R. Chaligne , I. Masilionis , E. Fontana , D. Ponce , C. Cho , A. Bush , L. Hill , N. Chao , A. D. Sung , S. Giralt , E. H. Vidal , et al., Sci. Transl. Med. 2022, 14, eabj2829. 35613281 10.1126/scitranslmed.abj2829 PMC9893439

[83] D. Lee , Z. S. Dunn , W. Guo , C. J. Rosenthal , N. E. Penn , Y. Yu , K. Zhou , Z. Li , F. Ma , M. Li , T.‐C. Song , X. Cen , Y.‐R. Li , J. J. Zhou , M. Pellegrini , P. Wang , L. Yang , Nat. Commun. 2023, 14, 6942. 37938576 10.1038/s41467-023-42619-2 PMC10632431

[84] C. Peters , L. Kouakanou , H. H. Oberg , D. Wesch , D. Kabelitz , Methods Enzymol. 2020, 631, 223. 31948549 10.1016/bs.mie.2019.07.019

[85] L. Kouakanou , Y. Xu , C. Peters , J. He , Y. Wu , Z. Yin , D. Kabelitz , Cell. Mol. Immunol. 2020, 17, 462. 31171862 10.1038/s41423-019-0247-8 PMC7192840

[86] H. Li , C. D. Pauza , Cancer Immunol. Immunother. 2011, 60, 361. 21107834 10.1007/s00262-010-0945-7 PMC3077899

[87] T. Tang , X. Huang , G. Zhang , Z. Hong , X. Bai , T. Liang , Signal. Transduct. Target. Ther. 2021, 6, 72. 33608497 10.1038/s41392-020-00449-4 PMC7896069

[88] N. Zacharakis , H. Chinnasamy , M. Black , H. Xu , Y.‐C. Lu , Z. Zheng , A. Pasetto , M. Langhan , T. Shelton , T. Prickett , J. Gartner , L. Jia , K. Trebska‐McGowan , R. P. Somerville , P. F. Robbins , S. A. Rosenberg , S. L. Goff , S. A. Feldman , Nat. Med. 2018, 24, 724. 29867227 10.1038/s41591-018-0040-8 PMC6348479

[89] T. Li , J. Fu , Z. Zeng , D. Cohen , J. Li , Q. Chen , B. Li , X. S. Liu , Nucleic Acids Res. 2020, 48, W509. 32442275 10.1093/nar/gkaa407 PMC7319575

[90] J. Liu , T. Lichtenberg , K. A. Hoadley , L. M. Poisson , A. J. Lazar , A. D. Cherniack , A. J. Kovatich , C. C. Benz , D. A. Levine , A. V. Lee , L. Omberg , D. M. Wolf , C. D. Shriver , V. Thorsson , H. Hu , S. J. Caesar‐Johnson , J. A. Demchok , I. Felau , M. Kasapi , M. L. Ferguson , C. M. Hutter , H. J. Sofia , R. Tarnuzzer , Z. Wang , L. Yang , J. C. Zenklusen , J. (J.). Zhang , S. Chudamani , J. Liu , L. Lolla , et al., Cell 2018, 173, 400.29625055

[91] P. F. Robbins , Y. F. Li , M. El‐Gamil , Y. Zhao , J. A. Wargo , Z. Zheng , H. Xu , R. A. Morgan , S. A. Feldman , L. A. Johnson , A. D. Bennett , S. M. Dunn , T. M. Mahon , B. K. Jakobsen , S. A. Rosenberg , J. Immunol. 2008, 180, 6116. 18424733 10.4049/jimmunol.180.9.6116 PMC2424230

[92] T. Chen , Xu Chen , S. Zhang , J. Zhu , B. Tang , A. Wang , L. Dong , Z. Zhang , C. Yu , Y. Sun , L. Chi , H. Chen , S. Zhai , Y. Sun , Li Lan , X. Zhang , J. Xiao , Y. Bao , Y. Wang , Z. Zhang , W. Zhao , Genomics Proteomics Bioinformatics 2021, 19, 578. 34400360 10.1016/j.gpb.2021.08.001 PMC9039563

[93] Database Resources of the National Genomics Data Center, China National Center for Bioinformation in 2023 , Nucleic Acids Res. 2023, 51, D18. 36420893 10.1093/nar/gkac1073 PMC9825504

