# Supplementary material for: Autologous Peripheral Vγ9Vδ2 T Cell Synergizes with αβ T Cell Through Antigen Presentation and BTN3A1 Blockade in Immunotherapy of Cervical Cancer
Source: Adv Sci (Weinh). 2025 Mar 17;12(18):2401230. doi: 10.1002/advs.202401230 (PMC12079532; doi:10.1002/advs.202401230)
Supplement: Supplementary file 1 — Supporting Information [file ADVS-12-2401230-s001.docx]

Supporting Information

**Autologous peripheral Vγ9Vδ2 T cell synergizes with αβ T cell through antigen presentation and BTN3A1 blockade in immunotherapy of cervical cancer**

*Min Wu^1,2#^, Jian Liu^1#^, Liting Liu^2#^, Yifan Yang^1^, Hong Liu^2^, Long Yu^3^,* *Haihong Zeng^4^, Shuo Yuan^2^, Ruiyi Xu^2^, Hangyu Liu^4^, Han Jiang^4^, Shen Qu^4^, Liming Wang^2^, Ying Chen^1^, Jingyu Wang^1^, Yuwei Zhang^4^, Shan He^4^, Ling Feng^1^, Junyan Han^5^, Wanjiang Zeng^6^*, Hui Wang^7,8^*, Yafei Huang^9,10^**

^#^Min Wu, Jian Liu and Liting Liu contributed equally to this work.

^1^Department of Obstetrics and Gynecology, Tongji Hospital and School of Basic Medicine, Tongji Medical College, Huazhong University of Science and Technology, Wuhan, China.

^2^Department of Gynecologic Oncology, Women’s Hospital, Zhejiang University School of Medicine, Hangzhou, Zhejiang, China.

^3^Beckman Coulter Commercial Enterprise (China) Co., Ltd, Shanghai, China.

^4^Department of Pathogen Biology, School of Basic Medicine, Tongji Medical College, Huazhong University of Science and Technology, Wuhan, China.

^5^Department of Immunology, School of Basic Medicine, Tongji Medical College, Huazhong University of Science and Technology, Wuhan, China.

^6^Department of Obstetrics and Gynecology, Tongji Hospital, Tongji Medical College, Huazhong University of Science and Technology, Wuhan, China. wjzeng@tjh.tjmu.edu.cn

^7^Department of Obstetrics and Gynecology, Tongji Hospital, Tongji Medical College, Huazhong University of Science and Technology, Wuhan, China. Cancer Biology Research Center (Key Laboratory of the Ministry of Education), Tongji Hospital, Tongji Medical College, Huazhong University of Science and Technology, Wuhan, China.

^8^Department of Gynecologic Oncology, Women’s Hospital, Zhejiang University School of Medicine, Hangzhou, Zhejiang, China. Zhejiang Provincial Key Laboratory of Precision Diagnosis and Therapy for Major Gynecological Diseases, Women’s Hospital, Zhejiang University School of Medicine, Hangzhou, Zhejiang, China.

^9^Department of Pathogen Biology, School of Basic Medicine, Tongji Medical College, Huazhong University of Science and Technology, Wuhan, China.

^10^State Key Laboratory for Diagnosis and Treatment of Severe Zoonotic Infectious Diseases, Huazhong University of Science and Technology, Wuhan, China.

**Corresponding authors:**

Hui Wang,

Department of Obstetrics and Gynecology, Tongji Hospital, Tongji Medical College, Huazhong University of Science and Technology, Wuhan, China. Cancer Biology Research Center (Key Laboratory of the Ministry of Education), Tongji Hospital, Tongji Medical College, Huazhong University of Science and Technology, Wuhan, China.

Department of Gynecologic Oncology, Women’s Hospital, Zhejiang University School of Medicine, Hangzhou, China. Zhejiang Provincial Key Laboratory of Precision Diagnosis and Therapy for Major Gynecological Diseases, Women’s Hospital, Zhejiang University School of Medicine, Hangzhou, Zhejiang, China.

Email: wang71hui@zju.edu.cn

Yafei Huang

Department of Pathogen Biology, School of Basic Medicine, Tongji Medical College and State Key Laboratory for Diagnosis and Treatment of Severe Zoonotic Infectious Diseases, Huazhong University of Science and Technology, Wuhan, Hubei, China

Email: huangy2018@hust.edu.cn

Wanjiang Zeng

Department of Obstetrics and Gynecology, Tongji Hospital, Tongji Medical College, Huazhong University of Science and Technology, Wuhan, China.

Email: wjzeng@tjh.tjmu.edu.cn

**Figure S1. Intratumoral Vδ1 and Vγ9Vδ2 T cells are associated with better prognosis and the infiltration of antitumor immune cells in CC. a**, Relationship of *TRDV1* expression level in tumor with CC patient survival in TCGA dataset (n = 306, Cox regression). **b**, **c**, The correlation of *TRGV9* (**b**) and *TRDV2* (**c**) expression levels with the enrichment of various immune cell subsets in cervical cancer from TCGA dataset. ssGSEA algorithm and spearman statistics were used. **d**, The correlation of *TRDV1* expression level with the indexes of ImmunoScore, ESTIMATEScore and StromalScore in cervical cancer from TCGA dataset (n = 306). Estimate algorithm [1.0.13] and spearman statistics were used. **e**, The correlation of *TRDV1* expression level with the infiltration of various immune cell subsets in cervical cancer from TCGA dataset. ssGSEA algorithm and spearman statistics were used. **f**, The correlation of *TRDV1* expression level with the expression of molecules associated with T cell activation, differentiation, chemotaxis and exhaustion in cervical cancer from TCGA dataset. Spearman statistics were used. **g**, The correlation analysis between the frequencies of Vδ1 γδ T subset and αβ T cell in tumor tissues of CC patients (n = 20). Pearson correlation and two-tailed *p* value were used.

**Figure S2. Peripheral Vγ9Vδ2 T cells from CC patients are higher in activation level and antigen presentation potential than those from HC. a**, The differentiation status of peripheral Vγ9Vδ2 T cells from CC patients and HC subjects. HC, n = 10. CC, n = 20. **b**, The volcanic graph of the differential expressed genes of pVγ9Vδ2 T cells between CC and HC. n = 2 per group. **c**, Gene set enrichment analysis of the up-regulated genes in pVγ9Vδ2 T cells from CC compared with those from HC. n = 2 per group. **d**, Gene set enrichment analysis of the corresponding up-regulated genes from GSE3982 analysis in pVγ9Vδ2 T cells from CC compared with those from HC. n = 2 per group. **e**, The protein expression levels (mean fluorescence Intensity, MFI) of molecules related to T cell activation, killing, antigen presentation, exhaustion and cytokines in pVγ9Vδ2 T cells from CC and HC. n = 10 per group. Error bars represent mean with SD in (a) and (e). *p* values were calculated using two-tailed Student’s *t* test in (a) and (e). **p* < 0.05; ***p* < 0.01; ****p* < 0.001. NS, Not Significant.

**Figure S3. The differentiation status and activation levels of the *in vitro* expanded pVγ9Vδ2 T cell products from CC and HC. a**, The percentage and purity of peripheral γδ T cells and Vγ9Vδ2 subset from CC patients and HC subjects before and after in vitro expansion were determined by flow cytometry. Total cell number of γδ T cells and the frequency of Vδ1 T cell subset in the in vitro expanded cell products from CC and HC were shown. HC, n = 10. CC, n = 20. **b**, The frequency of Vγ9 and Vδ2 double positive subset in the *in vitro* expanded cell products from CC and HC. n = 10 per group. **c**, The differentiation status of the *in vitro* expanded pVγ9Vδ2 T cells from CC and HC. n = 5 per group. **d**, The transcription levels of cytokines, chemokines and chemokine receptors in the *in vitro* expanded cell products from CC and HC. n = 2 per group. **e**, The protein expression levels of molecules related to T cell chemotaxis and NK cell activation in CC and HC derived pVγ9Vδ2 T cells before and after expansion for indicated days. n = 5 per group.

**Figure S4. Expanded autologous pVγ9Vδ2 T cells induce CC cell death by directly killing and CD4^+^ and CD8^+^ αβ** **T cells activation through antigen presentation. a**, Expanded pVγ9Vδ2 T cells from CC and HC were cocultured with CaSki, HeLa or SiHa cells in indicated ratios (T cell: cancer cell) for 6 hours, the cytotoxicity of T cells was determined by LDH assays. n = 5 independent patients. **b**, Expanded pVγ9Vδ2 T cells from CC patients were cocultured with DQ-OVA in the presence or absence of chloroquine (CQ) for 6 hours. The proportion of DQ fluorescence positive cells and the intensity of DQ fluorescence were determined by flow cytometry. **c**, Expanded pVγ9Vδ2 T cells from CC patients were cocultured with DQ-OVA for 6 hours. HLA-DR, CD40, CD80 and CD86 on pVγ9Vδ2 T cells were determined by flow cytometry. **d**, Mart-1_27-35_ specific TCR-T cells or control T cells (Pan-T) were cocultured with PHA, control pVγ9Vδ2 T cells or pVγ9Vδ2 T cells loaded with Mart-1_27-35_ peptide for 24 hours, the proportions of 4-1BB^+^ and OX40^+^ T cells were determined by flow cytometry. **e**, Mart-1_27-35_ specific TCR-T cells or control T cells (Pan-T) were cocultured with PHA, pVγ9Vδ2 T cells transduced with control vector or Mart-1_27-35_ overexpression vector for 24 hours, the proportions of 4-1BB^+^ and OX40^+^ T cells were determined by flow cytometry. **f**, Naïve αβ T cells were cocultured with control pVγ9Vδ2 T cells or CCLs-loaded pVγ9Vδ2 T cells for 72 hours, the proportions of 4-1BB^+^ and OX40^+^ T cells were determined by flow cytometry.

**Figure S5. Expanded pVγ9Vδ2 T cells induce CD4^+^ and CD8^+^ αβ** **T cells activation through antigen presentation. a**, PBMC from CC patient or HC was stimulated *in vitro* for pVγ9Vδ2 T cells expansion. Autologous naïve αβ T cells (isolated by Naive pan T cell isolation kit, 130-097-095, Miltenyi Biotec) were cocultured with control pVγ9Vδ2 T cells or CCLs-loaded pVγ9Vδ2 T cells for 120 hours in the presence of 200 IU/ml rhIL-2, the proportions of IFN-γ^+^, TNF^+^ and Granzyme B^+^ T cells were determined by flow cytometry. Mon, monocyte. Ctl, control. CCLs, cervical cancer cell lysates. **b**, PBMC from CC patient was stimulated *in vitro* for Vγ9Vδ2 T cells expansion. Autologous naïve αβ T cells were cocultured with control pVγ9Vδ2 T cells or CCLs-loaded pVγ9Vδ2 T cells for 120 hours in the presence of 1000 IU/ml rhIL-2, then the stimulated αβ T cells were purified and collected for subsequent cytotoxicity assays. Ctl, control. CCLs, cervical cancer cell lysates.

**Figure S6. Expanded pVγ9Vδ2 T cells induce αβ** **TIL activation through antigen presentation. a**, Establishment of tumor neoantigen prediction and validation system in CC patient. Total RNA and DNA were extracted from tumor tissues and peripheral blood of CC patients. RNA sequencing and whole exon sequencing (WES) were performed respectively. The sequencing results, patient HLA information and prediction tools were used to analyze and predict tumor neoantigens of patients, and a total of 12 long peptides containing 29 amino acids were obtained. In addition, αβ TILs were cultured and expanded using tumor tissues from CC patient CAT039 *in vitro*. pVγ9Vδ2 T cells were expanded using PBMCs from the same patient *in vitro*, and EBV-LCL were obtained by transfection of PBMC with EBV. Finally, pVγ9Vδ2 T cells or EBV-LCL were loaded with predicted neoantigen peptides and co-cultured with αβ TIL. αβ TIL activation and IFN-γ production were detected by flow cytometry and ELISPOT 24 hours later. M, monocyte. LCL, Epstein-Barr virus transformed lymphoblastoid cell line. **b**, αβ TILs were expanded from tumors of CC patient CAT039 and cocultured with control EBV-LCL or EBV-LCL loaded with predicted neoantigen single peptide (P1-P12) for 24 hours, the abundance of released IFN-γ was then determined by ELISPOT assay. P, peptide. **c**, αβ TILs derived from CC patient CAT039 were expanded and cocultured with control pVγ9Vδ2 T cells or pVγ9Vδ2 T cells loaded with predicted neoantigen single peptide (P1-P12) for 24 hours, and αβ TIL activation was determined by flow cytometry. P, peptide.

**Figure S7. Vγ9Vδ2/αβ T cell combinatorial therapy effectively inhibits cervical cancer growth. a**, In the SiHa-CDX models, HE staining of heart, liver, lung and kidney tissues of each group were analyzed. **b**, **c**, After the indicated treatments, tumor volume (**b**) and tumor images of each group (**c**) were analyzed (n = 4 mice/group). The statistics in the tumor volume were calculated with the day 37 data. **d**, **e**, In the SiHa-CDX models, single cell suspensions of blood and spleen were prepared. Human-derived CD45^+^ cells in the blood and spleen of each group were analyzed by flow cytometry (n = 4 mice/group). **f**, **g**, In the SiHa-CDX models, single cell suspensions of tumor tissues were prepared. The differentiation status of tumor-infiltrating CD4^+^, CD8^+^ and γδ T cells in tumor tissues **(f)**, and IFN-γ- and TNF-α-producing CD4^+^, CD8^+^ and γδ T cells in tumor tissues **(g)** were analyzed by flow cytometry (n = 4 mice/group). **h**, In the cervical cancer PDX tumor models, activated T cells (HLA-DR^+^), Vδ2^+^ T cell, CD4^+^ T cell, CD8^+^ T cell and CD107a^+^ cells in the tumor tissues were analyzed by fluorescence microscopy. Scale bar: 50 μm. Error bars represent mean with SD. *p* values were calculated using two-tailed Student’s *t* test. Survival curves were calculated with Log-rank (Mantel-Cox) test. **p* < 0.05; ***p* < 0.01; ****p* < 0.001. NS, Not Significant.

**Figure S8. Vγ9Vδ2/αβ T cell combinatorial therapy plays a synergistic effect with chemotherapy in the treatment of CC. a**, The expression levels of HLA-DR, CXCR3 and PD-1 on the surface of tumor-infiltrating Vδ2 T cell of CC patients with or without neoadjuvant chemotherapy were determined by flow cytometry. n = 9 per group. **b**, The proportion of peripheral Vδ1 T cell in CC patients with or without neoadjuvant chemotherapy were determined by flow cytometry. CC w/o chemo, n = 20. CC with chemo, n = 17. **c**, The expression levels of HLA-DR, CXCR3 and PD-1 on the surface of peripheral Vδ2 T cell of CC patients with or without neoadjuvant chemotherapy were determined by flow cytometry. n = 10 per group. **d**, The transcriptional levels of molecules related to T cell activation, differentiation, killing, exhaustion and antigen presentation in peripheral Vγ9Vδ2 T cell of CC patients with or without neoadjuvant chemotherapy. **e**, **f**, Experimental SiHa-CDX models were established. After the indicated treatments, tumor volume (**e**) and tumor images of each group (**f**) were analyzed (n = 4 mice/group). The statistics in the tumor volume were calculated with the day 37 data. **g**, **h**, In the SiHa-CDX models, single cell suspensions of blood and spleen were prepared. Human-derived CD45^+^ cells in the blood and spleen of each group were analyzed by flow cytometry (n = 4 mice/group). **i**, **j**, In the SiHa cell tumor models, single cell suspensions of tumor tissues were prepared, tumor-infiltrating γδ T cells **(i)**, and IFN-γ- and TNF-α-producing CD4^+^, CD8^+^ and γδ T cells in tumor tissues **(j)** were analyzed by flow cytometry (n = 4 mice/group). **k**, In the SiHa-CDX models, HE staining of heart, liver, lung and kidney tissues of each group were analyzed. DDP, cis-platin. Error bars represent mean with SD. *p* values were calculated using two-tailed Student’s *t* test. Survival curves were calculated with Log-rank (Mantel-Cox) test. **p* < 0.05; ***p* < 0.01. NS, Not Significant.

**Figure S9. Expression levels of BTN3A1 in CC cell lines. a**, CaSki cells were co-cultured with either irradiation-inactivated Vγ9Vδ2 T cells or non-irradiated Vγ9Vδ2 T cells at a 1:1 ratio in vitro. After 24 hours, the proportion of dead CaSki cells (CD3^-^7AAD^+^) was assessed using flow cytometry. **b**, **c**, CaSki-BTN3A1^OE^ **(b)** or CaSki-BTN3A1^KO^ **(c)** cells were cultured with Mart-1_27-35_ specific TCR-T cells for 12 hours. Irradiated pVγ9Vδ2 T cell and/or TCR γδ-blocking antibody were added to the cultures in indicated conditions. The percentage of CaSki cell death was determined by flow cytometry (n = 3 per group). **d**, BTN3A1 surface expressions in CaSki-vector, CaSki-BTN3A1^OE^ and CaSki-BTN3A1^KO^ cells were determined by flow cytometry. **e**, BTN3A1 expressions in CC cell lines C33A, CaSki and human umbilical vein endothelial cell line HUVEC were determined by flow cytometry. **f**, BTN3A1 expressions in CC cell lines were determined by western blot. **g**, **h**, Naïve αβ T cells were cultured and stimulated by CD3 and CD28 mAbs. IgG-Fc (**g**) or K562-Vector (**h**) was added to the cultures in the indicated conditions. CD4^+^ and CD8^+^ T cells proliferation were determined by flow cytometry (n = 3 per group). **i**, BTN3A1 expressions in K562-vector and K562-BTN3A1^OE^ cells were determined by flow cytometry. Error bars represent mean with SD. *p* values were calculated using two-tailed Student’s t test. ****p* < 0.001. OE, overexpression. KO, knockout. mAb, monoclonal antibody.

**Figure S10. IFN-γ induces the expression of BTN3A1 in CC cells in the STAT1 dependent manner. a**, CaSki, HeLa and SiHa cells were cultured and stimulated by indicated concentrations of IFN-γ (ng/ml) for 24 hours. The percentage of BTN3A1^+^ cells was determined by flow cytometry (n = 3 per group). **b**, CaSki, HeLa and SiHa cells were cultured and stimulated by indicated concentrations of IFN-γ (ng/ml), in the absence or presence of STAT1, NF-κB or ERK1/2 inhibitor, for 24 hours. The percentage of BTN3A1^+^ cells and BTN3A1 intensity were determined by flow cytometry (n = 3 per group).
